# A new ESI-LC/MS approach for comprehensive metabolic profiling of phytocannabinoids in *Cannabis*

**DOI:** 10.1038/s41598-018-32651-4

**Published:** 2018-09-24

**Authors:** Paula Berman, Kate Futoran, Gil M. Lewitus, Dzmitry Mukha, Maya Benami, Tomer Shlomi, David Meiri

**Affiliations:** 1 Department of Biology, Technion-Israel Institute of Technology, Haifa, 3200003 Israel; 2 Department of Computer Science, Technion-Israel Institute of Technology, Haifa, 3200003 Israel

## Abstract

Most clinical studies of *Cannabis* today focus on the contents of two phytocannabinoids: (-)-Δ^9^-*trans*-tetrahydrocannabinol (Δ^9^-THC) and cannabidiol (CBD), regardless of the fact that the plant contains over 100 additional phytocannabinoids whose therapeutic effects and interplay have not yet been fully elucidated. This narrow view of a complex *Cannabis* plant is insufficient to comprehend the medicinal and pharmacological effects of the whole plant. In this study we suggest a new ESI-LC/MS/MS approach to identify phytocannabinoids from 10 different subclasses, and comprehensively profile the identified compounds in diverse medical *Cannabis* plants. Overall, 94 phytocannabinoids were identified and used for profiling 36 of the most commonly used *Cannabis* plants prescribed to patients in Israel. In order to demonstrate the importance of comprehensive phytocannabinoid analysis before and throughout medical *Cannabis* clinical trials, treatments, or experiments, we evaluated the anticonvulsant effects of several equally high-CBD *Cannabis* extracts (50% w/w). We found that despite the similarity in CBD contents, not all *Cannabis* extracts produced the same effects. This study’s approach for phytocannabinoid profiling can enable researchers and physicians to analyze the effects of specific *Cannabis* compositions and is therefore critical when performing biological, medical and pharmacological-based research using *Cannabis*.

## Introduction

The *Cannabis* plant and its products consist of an enormous variety of chemicals from several natural product classes^1^. Among all the chemicals in *Cannabis*, C21 terpenophenolic cannabinoids are unique to this plant. Cannabinoids found in *Cannabis* are also termed phytocannabinoids, to distinguish this group of constituents from synthetic cannabinoids and endocannabinoids (the mammalian chemically endogenous cannabinoid receptor ligands).

Cannabinoids are being explored as pharmaceutical targets with potential applications in the treatment of anorexia, emesis, pain, inflammation, multiple sclerosis, neurodegenerative disorders, epilepsy, cancer, cardiovascular disorders, and more^2–11^. Their pharmacological effects have been found to be mediated mainly through two specific plasma membrane G-protein-coupled receptors, referred to as cannabinoid receptors CB1 and CB2. Today, more receptors of the endocannabinoid system are known, including GPR55, GPR119 and TRPV1, however their physiological functions are still mostly unknown^12–16^. In addition it is widely accepted today that different cannabinoids act on multiple targets^17,18^.

According to a recent review^19^, out of more than 545 metabolic constituents identified from *Cannabis*^20^, 144 have been isolated and identified as phytocannabinoids. Phytocannabinoids have been conventionally classified into the following 11 subclasses according to their chemical structures: (1) cannabigerol (CBG, **1**); (2) (-)-Δ^9^-*trans*-tetrahydrocannabinol (Δ^9^-THC, **2**); (3) cannabidiol (CBD, **3**); (4) cannabichromene (CBC, **4**); (5) cannabinol (CBN, **5**); (6) (-)-Δ^8^-*trans*-tetrahydrocannabinol (Δ^8^-THC, **6**); (7) cannabicyclol (CBL, **7**); (8) cannabinodiol (CBND, **8**); (9) cannabielsoin (CBE, **9**); (10) cannabitriol (CBT, **10**); and (11) miscellaneous types^1,20^.

**Figure 1:**
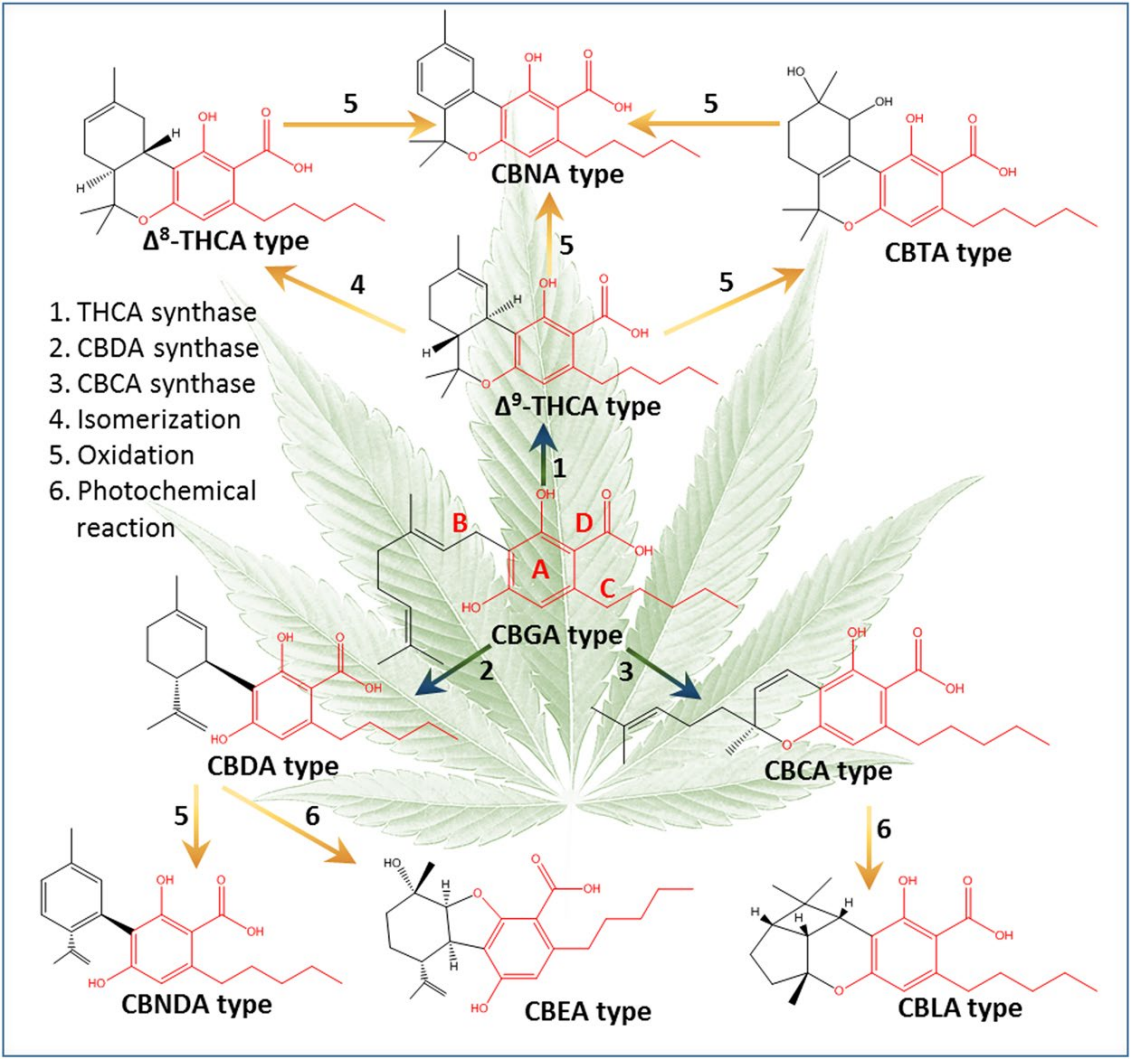
Phytocannabinoid biosynthesis and degradation routes and products according to the literature^19–28^. The most prevalent acid components are presented for each type of phytocannabinoid.

Phytocannabinoids are biosynthesized as acids. Decarboxylation via heat produces the more familiar, neutral-types. The suggested biosynthesis and decomposition pathways of the different types of phytocannabinoid acids are summarized in Fig. 1^19–28^. In general, CBG, Δ^9^-THC, CBD and CBC phytocannabinoid subclasses are biosynthesized in *Cannabis* plants, while the remaining six subclasses are probably the result of decomposition either in the plant or due to poor storage conditions following harvest. All subclasses of phytocannabinoids derive initially from CBG-type ones, and therefore bear similarity in terms of chemical structures (marked in red on Fig. 1). Cannabigerolic acid (CBGA) consists of (A) a resorcinol (benzene-1,3-diol) ring, (B) a terpene moiety, (C) an alkyl chain, and (D) a carboxylic group (Fig. 1). (-)-*trans*-Δ^9^-tetrahydrocannabinolic acid (Δ^9^-THCA, **11**), cannabidiolic acid (CBDA, **12**), and cannabichromenic acid (CBCA, **13**) differ only by the enzymatic cyclization of the terpene moiety. Cannabinolic acid (CBNA, **14**) and cannabinodiolic acid (CBNDA, **15**) are the fully aromatized forms of Δ^9^-THCA and CBDA, respectively. In (-)-*trans*-Δ^8^-tetrahydrocannabinolic acid (Δ^8^-THCA, **16**) the double bond at the ninth carbon isomerizes to the eighth position. Cannabicyclolic acid (CBLA, **17**) is formed by the creation of two additional rings to CBCA. Cannabielsoic acid (CBEA, **18**) is the result of hydroxylation and the attachment of one of the two phenolic oxygen atoms at the endocyclic double bond of the monoterpene unit of CBDA. (±)-*trans*/*cis*-cannabitriolic acid (CBTA, **19**) isomers are the hydroxylated forms of Δ^9^-THCA with two additional hydroxyl groups at different positions.

*Cannabis* strains significantly vary in their chemical compositions. The concentration of *Cannabis*’s compounds depends on the plant’s tissue-type, age, variety, growth conditions (nutrition, humidity and light levels), harvest time, and storage conditions^17,29^. Analyzing the chemical content of the plants is of major importance considering that the concentrations of these constituents and their interplay may determine medicinal effects and adverse side effects.

The most common method used for the analysis of phytocannabinoids in *Cannabis* extracts is gas chromatography (GC), either coupled to a flame ionization detector or a mass-spectrometer (MS). However, there are three notable limitations for this approach^30,31^: (1) Injection of *Cannabis* extracts for analysis by GC typically results in decarboxylation in the injection port, and consequently it is only the decarboxylated phytocannabinoids that are measured directly by these techniques. (2) While derivatization of the metabolite extract via silylation enables the measurement of both acid and neutral phytocannabinoids, a complete derivatization yield is difficult to obtain and thus quantification is less reliable. (3) It has also been suggested that phytocannabinoids may thermally degrade (oxidize, isomerize) in the injector port and column.

Utilizing high performance liquid chromatography (HPLC) instead of GC resolves the threat of phytocannabinoid decomposition as a result of heating^30,31^. Moreover, in contrast to the developed methods for phytocannabinoid identification by GC, HPLC can be directly applied for fractionation of extracts into separate pure compounds in preparative devices. Fractionation of extracts is highly important in medical research and for structure elucidation.

Although there is a great diversity in chemical constituents between *Cannabis* strains and the number of phytocannabinoids is large, most studies that profile phytocannabinoids from *Cannabis* report only the major phytocannabinoids in the extract, usually up to the eight most common components^32–38^. This is partly due to the fact that there are currently only a limited number of phytocannabinoid analytical standards commercially available. In this study, we present an approach for comprehensive identification and quantification of phytocannabinoids in *Cannabis* using liquid chromatography mass spectrometry (LC/MS) as presented in Supplementary Fig. S1.

## Results

### LC-MS/MS analysis of available phytocannabinoid standards

**Figure 2:**
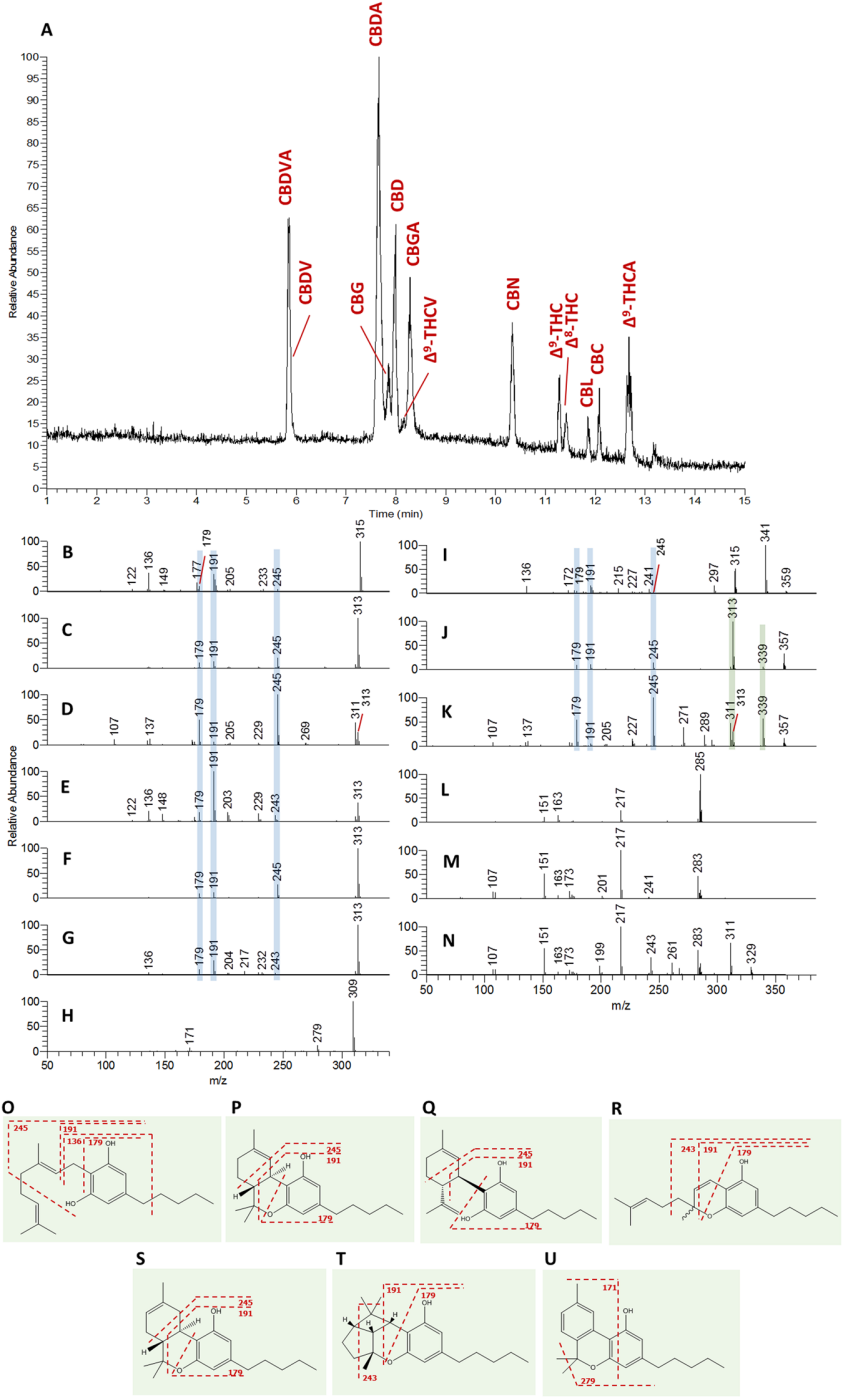
Observed chromatographic and MS characteristics of phytocannabinoid standard materials. (**A**) Total ion current (TIC) chromatogram of the 13 available phytocannabinoid standards, and their MS/MS spectra according to (**B**) CBG, (**C**) Δ^9^-THC, (**D**) CBD, (**E**) CBC, (**F**) Δ^8^-THC, (**G**) CBL, (**H**) CBN, (**I**) CBGA, (**J**) Δ^9^-THCA, (**K**) CBDA, (**L**) Δ^9^-THCV, (**M**) CBDV, and (**N**) CBDVA. Fragmentation structures for (**O**) CBG, (**P**) Δ^9^-THC, (**Q**) CBD, (**R**) CBC, (**S**) Δ^8^-THC, (**T**) CBL, and (**U**) CBN were determined according to the observed MS/MS spectra of each phytocannabinoid. Values of m/z in this figure are presented as nominal masses to improve interpretation of spectra. Accurate masses for the main fragments appear in Supplementary Figs S3–S9. The blue and green highlights mark identical m/z fragments for the neutral and acid phytocannabinoids.

The total ion current (TIC) chromatogram of the 13 phytocannabinoid standards is shown in Fig. 2A. According to this chromatogram, the observed relative order of elution of the available pentyl neutral phytocannabinoid analytical standards from the reversed phase (RP) column is as follows: CBG, CBD, CBN, Δ^9^-THC, Δ^8^-THC, CBL and CBC. This order of elution can be explained by decreasing molecular lipophilicity according to the decreasing number of hydroxyl groups on the aromatic ring of each component (two hydroxyls for CBG and CBD, and one hydroxyl for Δ^9^-THC and CBC), and ring opening leading to an additional long aliphatic chain on CBC in relation to Δ^9^-THC. The effect of hydroxylation on lipophilicity of natural and synthetic phytocannabinoids was demonstrated by Thomas *et al*.^39^ which concluded that the introduction of a single hydroxyl group to Δ^9^-THC decreased lipophilicity 3- to 40-fold, depending on the site of attachment. Compared to Δ^9^-THC, CBN elutes faster probably due to a decrease in lipophilicity with the addition of two double bonds, and Δ^8^-THC is slightly more retained due to the double bond isomerization (Fig. 2A). CBL elutes prior to CBC possibly due to a decrease in lipophilicity and increase of steric hindrance by two additional rings (Fig. 2A).

(-)-Δ^9^-tetrahydrocannabivarin (Δ^9^-THCV, **20**) and cannabidivarin (CBDV, **21**), the propyl homologues of Δ^9^-THC and CBD, respectively, have shorter retention times compared to their respective pentyl phytocannabinoids as a result of decreasing lipophilicity with shorter chain lengths. CBGA and Δ^9^-THCA, the acid precursors of CBG and Δ^9^-THC, respectively, have longer retention times; whereas CBDA and cannabidivarinic acid (CBDVA, **22**), the acid precursors of CBD and CBDV elute before the respective neutral ones.

The MS/MS spectra for the 13 standards at a normalized collision energy (NCE) of 40 appear in Fig. 2B–N. Plots of the ion abundance of specific deprotonated precursor and product ions as a function of NCE for all the available neutral pentyl and propyl phytocannabinoids appear in Supplementary Fig. S2A–I. CBG has a distinct mass (315.2330 Da in deprotonated form) that is different from all other types of phytocannabinoids, which allows for definite and straightforward identification. According to its chemical structure, the product ions at *m/z* of 245.1547, 191.1078 and 179.1067 (Fig. 2B) can result from the cleavage of the terpene moiety at distinct positions such that their mass fragments include the resorcinol ring and alkyl chain (Fig. 2O). Cleavage of the alkyl chain of the product ion at *m/z* of 191.1078 yields the product ion at *m/z* of 136.0530.

Δ^9^-THC, CBD, CBC, Δ^8^-THC and CBL have similar chemical formulas and exact masses but their structures and fragmentation patterns are different (Fig. 2C–G). All of these phytocannabinoids exhibit the product ions at *m/z* 245.1547, 191.1078 and 179.1067 as also presented for CBG but with very different relative abundances. According to Supplementary Fig. S2B and E, each of these compounds displays specific fragmentation patterns, where the intensity of the precursor and major product ions varies with energy in a characteristic manner. For CBD the product ion at *m/z* of 311.2017 is apparently more stable than the molecular ion at *m/z* of 313.2173 (Fig. 2D). For CBC and CBL the product ion at *m/z* of 243.1391 is more predominant than 245.1547 (Fig. 2E,G, respectively). Δ^9^-THC and Δ^8^-THC have very similar fragmentation patterns up to a NCE of 40 (Supplementary Fig. S2B and E, respectively). Above this energy, the product ion at *m/z* of 191.1078 in relation to the precursor ion of *m/z* of 313.2173 can be used for differentiation between the two phytocannabinoids.

According to the observed accurate mass fragments in Fig. 2C–G we suggest the fragmentation structures in Fig. 2P–T, respectively. Interestingly, the CBL ion abundance profile as a function of NCE exhibits a very similar profile compared to that of CBC, only shifted to the right (Supplementary Fig. S2F and D, respectively), such that CBL at an NCE of 60 has an almost identical MS/MS spectrum as CBC at an NCE of 40. This suggests that fragmentation of CBL probably leads to the opening of the two additional rings, thereby creating a CBC-like structure (Fig. 2T).

CBN has a very simple mass spectrum due to the presence of two highly stable aromatic rings, and consists mainly of the molecular ion and two product ions at *m/z* of 279.1391 and 171.0815 (Fig. 2H). The suggested fragmentation structure for CBN (Fig. 2U) is based on the most likely sites of fragmentation that lead to the observed product ions.

According to CBGA, Δ^9^-THCA and CBDA analytical standards, acid phytocannabinoids exhibit very similar MS/MS spectra to their respective neutral compounds, with additional neutral losses of CO_2_ and H_2_O because of decarboxylation and dehydration (Fig. 2B–D and I–K, for the neutral and acid phytocannabinoids, respectively). Some minor additional product ions can be observed for the acid phytocannabinoids which result from fragments that include the carboxylic group.

In the case of alkyl homologues, similar fragmentation patterns can be observed for product ions with the appropriate mass decrements compared to the pentyl phytocannabinoids. For example, the product ions of Δ^9^-THCV at *m/z* of 217.1234, 163.0765 and 151.0765 show similar intensities as those of Δ^9^-THC at *m/z* of 245.1547, 191.1078 and 179.1067 (Fig. 2L,C, respectively). CBD and CBDV exhibit the same product ions (Fig. 2D,M, respectively), however, their intensities are inherently different than those of Δ^9^-THC and Δ^9^-THCV. In addition, the response of these product ions varies with energy in a characteristic manner for each type of phytocannabinoid, such that Δ^9^-THC and Δ^9^-THCV exhibit similar profiles but different ones than CBD and CBDV (Supplementary Fig. S2B and H for Δ^9^-THC and Δ^9^-THCV, respectively; and Fig. S2C and I for CBD and CBDV, respectively). This phenomenon of observed characteristic ion abundances versus energy for the same phytocannabinoid subclass was previously observed for a GC/MS analysis with electron impact ionization of a *Cannabis* extract, and was suggested as a tool for identification of unknown peaks in the GC/MS TIC of *Cannabis* extracts^40,41^. CBDVA exhibits the same MS/MS spectrum as CBDV with the additional neutral losses of CO_2_ and H_2_O as suggested for the carboxylic acid group (Fig. 2N,M, respectively).

### LC-MS/MS based detection of additional phytocannabinoids in *Cannabis* ethanolic extracts

**Figure 3:**
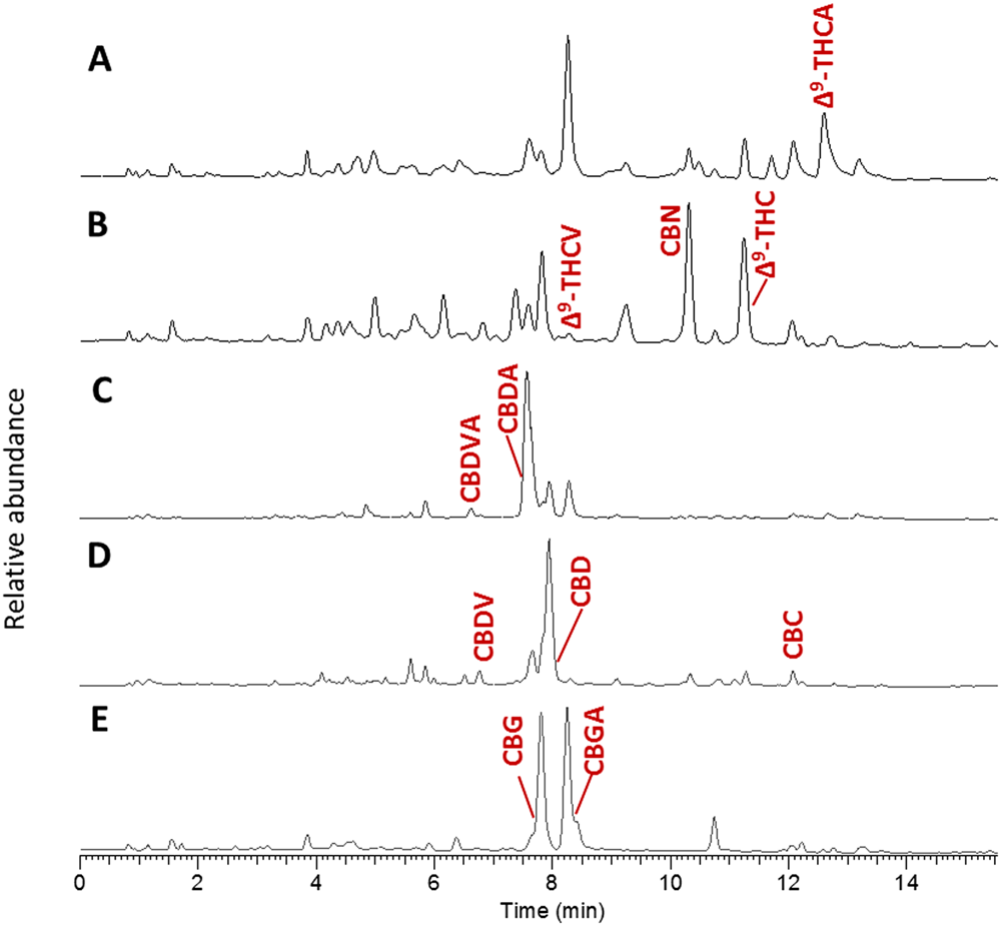
LC/MS TIC of (**A**) untreated and (**B**) decarboxylated high-Δ^9^-THCA; (**C**) untreated and (**D**) decarboxylated high-CBDA; and (**E**) partly decarboxylated high-CBGA *Cannabis* strains injected in the prepared concentration, with annotated phytocannabinoids identified according to analytical standards on the samples with the highest expression.

The LC/MS TIC chromatograms of untreated and decarboxylated high-Δ^9^-THCA and high-CBDA, and partly decarboxylated high-CBGA *Cannabis* strains injected in the extracted concentration without further dilution appear in Fig. 3A–E, respectively. As shown, many additional compounds can be observed compared to the LC/MS TIC chromatogram of the 13 phytocannabinoid standards in Fig. 2A. In order to identify additional phytocannabinoids in *Cannabis* for which analytical standards are not available we employed a data-dependent LC/MS/MS screen. The screen was performed by a compiled list of masses (Supplementary Table S1) according to (a) the isolated phytocannabinoids in the literature as reviewed by ElSohly and Gul^20^, and (b) additional potential phytocannabinoids that were added according to the biosynthesis and decomposition pathways described in the literature and summarized in Fig. 1^19–28^. Phytocannabinoids in this list are referred by the names in the literature^20^. The additional components were marked with an asterisk in Supplementary Table S1 and were named by common abbreviations following the nomenclature applied to conventional phytocannabinoids^1,20^. For example, we looked for ions of both acid and neutral phytocannabinoid compounds that can exist in the plant, even if they had not been previously isolated and reported in the literature. This was the case, for example, for CBNDA and CBTA phytocannabinoids in Fig. 1. Also, if methyl, propyl and butyl-phytocannabinoids of Δ^9^-THC ((-)-Δ^9^-*trans*-tetrahydrocannabiorcol-C1 (Δ^9^-THCO, **23**), Δ^9^-THCV and (-)-Δ^9^-*trans*-tetrahydrocannabiorcol-C4 (Δ^9^-THC-C4, **24**), respectively) both neutral and acid, were previously identified^20^, we assumed that the same alkyl homologues exist for the CBG-type phytocannabinoids, from which they were biosynthesized. We also presumed that additional alkyl homologues may exist for all the other types of phytocannabinoids, following their biosynthesis and decomposition routes^42–44^. Other derivatives with hydroxyl and methyl functional groups were also considered for the different phytocannabinoid subclasses. Given that phytocannabinoids are biosynthesized in the plant in the acid form, and that the content of neutral components is naturally low, several common strains were decarboxylated prior to extraction in order to increase the abundance of neutral phytocannabinoids in the sample. This was done to improve our ability to identify them when establishing the LC/MS/MS spectral library (Supplementary Fig. S1).

In this study, we used the retention times and MS/MS fragmentation patterns of the available analyzed pentyl neutral phytocannabinoid standards as the reference for the identification of additional phytocannabinoid homologues from the same subclass, for which analytical standards are not available. Overall 40 additional phytocannabinoids from the seven phytocannabinoid subclasses were identified in *Cannabis* ethanolic extracts. The MS/MS spectra, names, retention times, accurate masses, and fragmentation structures for the identified phytocannabinoids from the CBG, Δ^9^-THC, CBD, CBC, CBN, Δ^8^-THC and CBL subclasses appear in Supplementary Fig. S3–S9, respectively.

All the identified alkyl homologues of a specific phytocannabinoid subclass elute from the RP column in the order C1 → C3 → C4 → C5, as a result of increasing lipophilicities with longer chain lengths. This order is also observed for their respective acids. For all alkyl homologues of a specific phytocannabinoid type, an appropriate *m/z* shift in the MS/MS spectra of all the product ions that include the alkyl chain is observed. Interestingly, the product ions at *m/z* of 136.0530 and 171.0815 for CBG and CBN, respectively, are observed in all their respective alkyl homologues (Supplementary Figs S3A and S7A, respectively). This information strengthens the fragment assignments of the product ions that undergo cleavage of the alkyl chain for CBG and CBN (Fig. 2O,U, respectively).

Methyl and hydroxyl derivatives of the different types of phytocannabinoids were identified, as presented in Supplementary Figs S3–S5 and S7. The hydroxylated and methylated compounds exhibit shorter and longer retention times, respectively, compared to the pentyl neutral phytocannabinoid standards. These compounds exhibit similar MS/MS spectra as the pentyl phytocannabinoid standards with the appropriate mass increments for the hydroxyl and methyl groups. Sesquicannabigerol (SesquiCBG, **25**) and sesquicannabigerolic acid (SesquiCBGA, **26**) from the CBG-type phytocannabinoids, were identified by a large increase in retention times (Supplementary Fig. S3C) and identical MS/MS spectra with shifts of the deprotonated molecular ions to *m/z* values of 383.2955 and 409.2748, compared to CBG and CBGA (Supplementary Fig. S3A and B, respectively).

Assignment of unidentified peaks in *Cannabis* ethanolic extracts to the remaining three phytocannabinoid subclasses (CBND, CBE and CBT), for which no standard materials were available was also performed, although identification for these components should be treated with more caution. Since no reference materials from the same subclass were available, the identification of the phytocannabinoids in these subclasses was performed by comparing their chromatographic and MS characteristics in relation to those of their precursors (Fig. 1) and by accurate masses. The MS/MS spectra, names, retention times, accurate masses and fragmentation structures for the identified CBND-, CBE- and CBT-type phytocannabinoids appear in Supplementary Figs S10–S12. As expected by the phytocannabinoid decomposition routes (Fig. 1), CBND, CBE, and CBT were most abundantly found in aged high-CBD and high-Δ^9^-THC *Cannabis* strains, respectively.

CBND is the aromatized form of CBD and it has a similar chemical formula and exact mass as CBN. CBND was found to elute before CBD (Supplementary Figs S5C and S10C for CBD and CBND, respectively), as suggested for the effect of the additional aromatic ring compared to CBD and as was proposed for the elution order of CBN in relation to Δ^9^-THC (Fig. 2A). In comparison to CBN, CBND also has a very simple MS/MS spectrum due to the presence of the two very stable aromatic rings, with mainly two product ions at *m/z* of 279.1391 and 171.0815 (Supplementary Fig. S10A). CBE also elutes prior to CBD (Supplementary Figs S5C and S11C, respectively). It exhibits a neutral loss of 18 Da due to cleavage of one hydroxyl group (Supplementary Fig. S11A). Other product ions include *m/z* of 271.1704, 205.1234 and 179.1067 according to Supplementary Fig. S11D.

CBT-type phytocannabinoids have several structural isomers whose definite assignment is difficult to determine without their isolation and structural elucidation. Still, several components were identified from this group according to their accurate masses, two neutral losses of 18 Da from the two additional hydroxyl groups, and additional product ions at *m/z* of 285.1860, 191.1078 and 179.1067 (Supplementary Fig. S12A). CBT-type phytocannabinoids elute faster than Δ^9^-THC due to a reduction in lipophilicity, as previously mentioned for hydroxyl groups.

Propyl homologues and acid precursors for the additional three phytocannabinoid subclasses were also identified following the same rules described above. In total 14 additional phytocannabinoids were identified from the last three phytocannabinoid subclasses.

Overall we established a spectral mass library for 67 identified phytocannabinoids from all 10 phytocannabinoid subclasses, as detailed in Supplementary Figs S3–S12. An additional 27 phytocannabinoids whose absolute identification could not be determined with certainty at the time of publication were also added to the spectral library for the purpose of quantification (Supplementary Fig. S13). According to the proposed minimum reporting standards for chemical analysis^45^, the identified phytocannabinoids in this library should be regarded by the following levels of confidence: (a) 13 phytocannabinoids (type I) were identified by analytical standards with the highest level of confidence; (b) 40 phytocannabinoids (type II) were putatively identified according to the characterized phytocannabinoid subclasses; (c) 14 phytocannabinoids (type III) were putatively identified via their chromatographic and spectral similarities between the remaining three subclasses and their precursors; and (d) an additional 27 phytocannabinoids (type IV) were attributed as potential phytocannabinoids by accurate mass and fragmentation patterns typical to the ones presented for the identified phytocannabinoids in this research. The absolute identification of type IV phytocannabinoids could not be determined with certainty, however they could still be differentiated and quantified based upon spectral data^45^.

### Quantification of phytocannabinoids

In the presented work, absolute quantification of type I phytocannabinoids in *Cannabis* extracts was performed by developing and validating external calibrations (Supplementary Table S2). The calibration curves were linear for all the analytes (R^2^ > 0.99). Limit of quantification (LOQ) for each component was determined as maximum deviation from expected concentrations of 20%, and minimum signal-to-noise ratios of 10. Precision was determined according to relative standard deviations (RSDs) for repeatability and reproducibility. Repeatability was quantified by intra-day variation from ethanolic extractions of the same sample (n = 5), and reproducibility by inter-day variation was verified via extracting the same sample in triplicates on three different days. Precision of Δ^9^-THCA, Δ^9^-THC, Δ^9^-THCV and CBN were quantified using a second sample. Δ^8^-THC and CBL were not observed in either of the samples.

In order to compare the performance of the external calibration method with the more work-intensive standard addition approach, we spiked a *Cannabis* sample with three different concentrations of standards. For each compound, the measured concentration with the standard addition was *z*-transformed using data (mean and standard deviation) acquired from replicates of external calibration measurements. The difference between the two experimental approaches was assessed by comparing the sum of squared *z*-scores to cumulative density functions of χ^2^ distribution with the corresponding degrees of freedom (see Methods section). The statistical test revealed no significant difference between the two approaches (p = 0.13).

For all other phytocannabinoids (types II-IV), accurate absolute quantification is difficult to achieve since no analytical standards were available, and their ionization efficiencies (IEs) were unknown. However, comparison of the observed responses for the same concentration for each of the available phytocannabinoid standards revealed some trends which were used for extrapolation of absolute concentrations for all other phytocannabinoids. These trends are described as follows: In general, acid phytocannabinoids (Δ^9^-THCA, CBDA, CBGA and CBDVA) exhibited larger IEs than their corresponding neutral ones (Δ^9^-THC, CBD, CBG and CBDV, respectively), and pentyl phytocannabinoids (Δ^9^-THC, CBD and CBDA) exhibited comparable IEs as propyl ones (Δ^9^-THCV, CBDV and CBDVA, respectively). Therefore, absolute concentrations of the neutral and acid type II phytocannabinoids were calculated using the calibration curves of the analytical standards from the same subclass. For the acid alkyl homologues of CBCA we chose the calibration curve of CBDA according to a resemblance in IEs between CBD and CBC. For the acid homologues of CBN-type phytocannabinoids, a calibration curve was developed by multiplying the observed peak areas of CBN by the average ratio of ion responses in the same concentration between the available neutral and acid pentyl phytocannabinoids. The calibration curves for types III and IV acid and neutral phytocannabinoids were set as the average ion response for the same concentration for all the available acid and neutral phytocannabinoid standards.

### Phytocannabinoid profiling of different medical *Cannabis* strains

In order to demonstrate the importance of phytocannabinoid profiling, we performed a differential analysis of medical *Cannabis* plants, by which samples were compared per phytocannabinoid to study the variation in the amounts and ratios of phytocannabinoids in medical *Cannabis* plants. To this end, 36 of the most commonly used *Cannabis* plants were chosen from over a hundred available strains prescribed to patients in Israel. Due to a large variation in concentrations of phytocannabinoids in *Cannabis* strains, all samples were injected and analyzed by LC/MS in three dilutions (1:9, 1:99 and 1:999 v/v *Cannabis* extract to ethanol). Identification of phytocannabinoids in each analysis was performed according to the accurate masses and retention time designations in Supplementary Figs S3–S13. Peak area values for each dilution which diverged from the linear dynamic range of each component were omitted from the analyses and the remaining areas were averaged.

**Figure 4:**
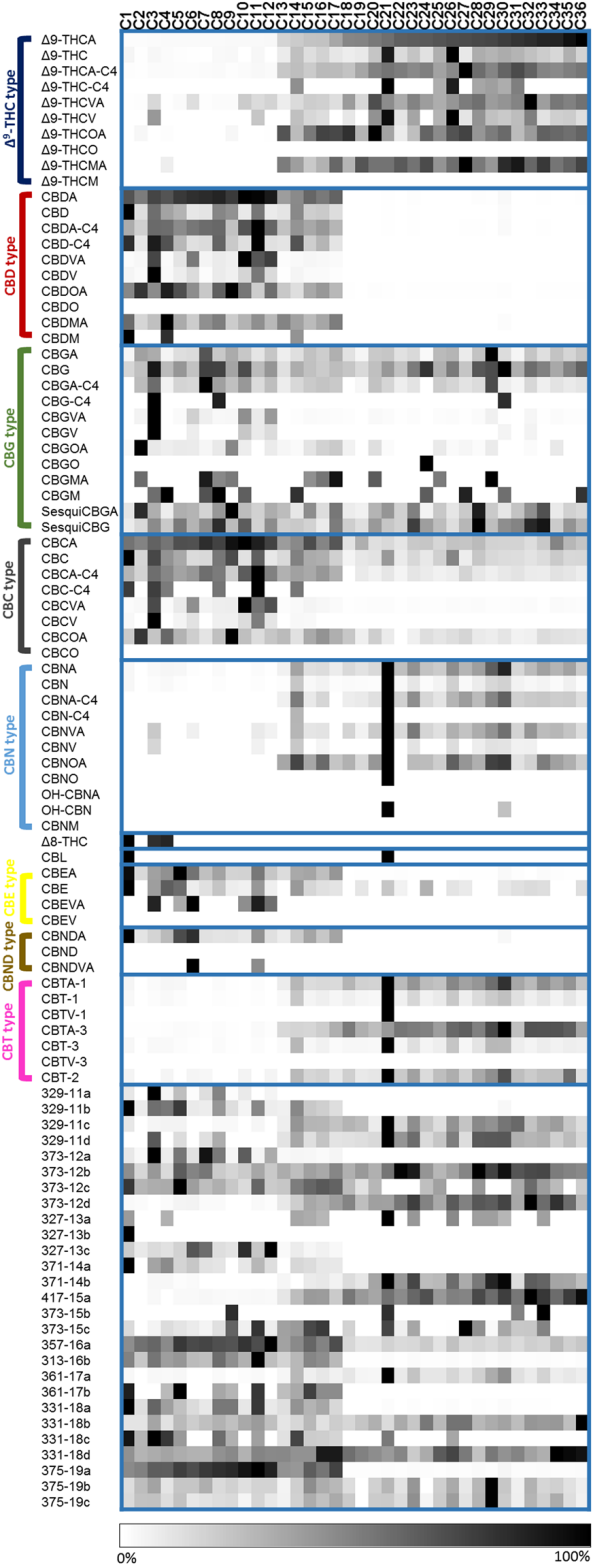
Phytocannabinoid profiling of 36 different medical *Cannabis* strains. The LC/MS concentrations of each phytocannabinoid were normalized to the highest value and compared per phytocannabinoid in a heat map. Strains were arranged according to increasing Δ^9^-THCA content (first line) and phytocannabinoids by subclasses.

The differential analysis of the 36 *Cannabis* samples (C1-C36) appears in Fig. 4. In this heat map, the LC/MS concentrations of each phytocannabinoid were normalized to the highest value and compared per phytocannabinoid. Strains were arranged according to increasing Δ^9^-THCA content, and phytocannabinoids by subclasses. The ranges of absolute concentrations for each phytocannabinoid for the 36 samples appear in Supplementary Table S3. Deviations of concentrations for the approximated values calculated according to the highest and smallest calibration curves of the type I phytocannabinoids were also calculated for types II-IV phytocannabinoids (Supplementary Table S3).

In Supplementary Fig. S14A we show a histogram of the percentage of phytocannabinoids which differ by more than 20% for every pair of samples (by the larger number). As shown, no two *Cannabis* samples had the same phytocannabinoid profile. According to this histogram, the concentrations of most pairs of samples differed by more than 71% of the phytocannabinoid components. Even among the most similar (to one another) *Cannabis* samples (C23 and C28), 30.6% of the phytocannabinoid components differed by more than 20%.

To study specific relationships between the phytocannabinoids, we used hierarchical clustering to order the heat map of both *Cannabis* samples and phytocannabinoids (Supplementary Fig. S14B). As expected by the decomposition routes in Fig. 1, CBN- and CBT-type phytocannabinoids, and CBND- and CBE-types were closely grouped with those of Δ^9^-THC- and CBD-types, respectively (Supplementary Fig. S14B). CBC-type phytocannabinoids were grouped with CBD-types. According to the observed clustering, *Cannabis* samples were divided into two main groups by the contents of Δ^9^-THC- (G1) and CBD-type (G2) phytocannabinoids and decomposition products (G2 also includes *Cannabis* strains with similar ratios of Δ^9^-THCA:CBDA).

**Figure 5:**
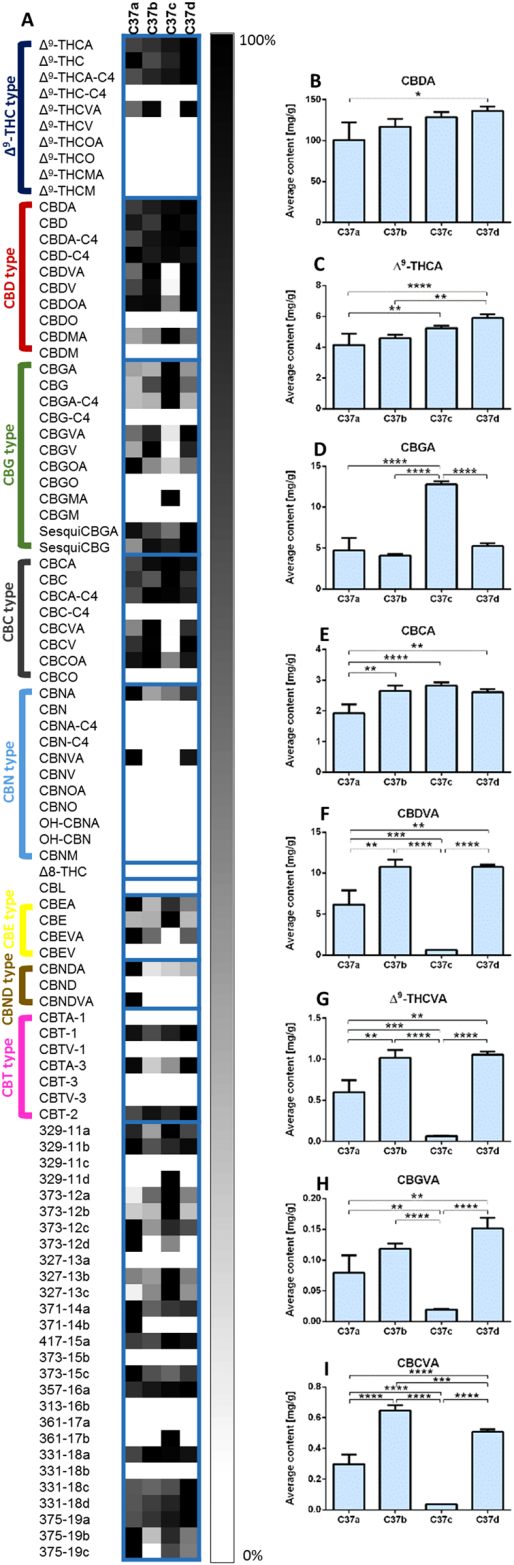
Phytocannabinoid profiling of a specific high-CBD *Cannabis* strain that is used to treat children with epilepsy. (**A**) LC/MS differential analysis of four samples from the same strain with the same genetics, planted and harvested in the same way and at the same time, but grown at four different greenhouses. Two samples were extracted from each plant and prepared in three dilutions (1:9, 1:99, and 1:999  v/v *Cannabis* extract to ethanol). Samples were injected to the LC/MS in duplicates. The average LC-MS concentrations of each phytocannabinoid were normalized to the highest value and compared per phytocannabinoid in a heat map. Strains were arranged according to increasing Δ^9^-THCA content (first line) and phytocannabinoids were arranged by subclasses. Quantitative comparison of the absolute concentrations of (**B**) CBDA, (**C**) Δ^9^-THCA, (**D**) CBGA, (**E**) CBCA, (**F**) CBDVA, (**G**) Δ^9^-THCVA, (**H**) CBGVA and (**I**) CBCVA, show significant differences between *Cannabis* samples from the same strain as a result of growing conditions. All values are reported as mean ± SD (one-way ANOVA, Tukey HSD post hoc test *p < 0.05, **p < 0.01, ***p < 0.001, ****p < 0.0001).

In order to further demonstrate the importance of comprehensive phytocannabinoid analysis we evaluated the phytocannabinoid profiles of specific high-CBD oil-based extracts that are being used today to treat refractory childhood epilepsy^7,8,46^. Generally, besides CBD and Δ^9^-THC contents, most other phytocannabinoids are usually not reported. As a first step we present in Fig. 5A a differential analysis of one specific high-CBD *Cannabis* strain. Here we compare the phytocannabinoid profile of this same strain with the same genetics (as all these plants came from the same “mother” plant), planted and harvested in the same way and at the same time, but grown in four different greenhouses. Quantitative comparisons of some pentyl and propyl phytocannabinoid acids appear in Fig. 5B–I. Indeed, these plants show very similar CBDA concentrations (significant differences were observed only for samples C37a and C37d, 100.8 ± 21.6 mg/g and 136.4 ± 10.0 mg/g, respectively, p < 0.05, Fig. 5B), but differ considerably in their content of other biosynthesized phytocannabinoids as a result of growing conditions (Fig. 5C–I). Most striking differences were observed for sample C37c, which showed significantly higher CBGA content compared to all other samples (p < 0.0001, Fig. 5D), and considerably lower contents of all propyl phytocannabinoids (p < 0.01, Fig. 5F–I for CBDVA, (-)-Δ^9^-tetrahydrocannabivarinic acid (Δ^9^-THCVA, **27**), cannabigerovarinic acid (CBGVA, **28**) and cannabichromevarinic acid (CBCVA, **29**), respectively). The neutral counterparts of several of the aforementioned phytocannabinoids and others have been recently suggested to contribute to the anticonvulsant effects of *Cannabis*, in particular CBDV but also Δ^9^-THCV and CBG^46^. This shows that even the same strain may differ widely as a result of differences in growing conditions.

**Figure 6:**
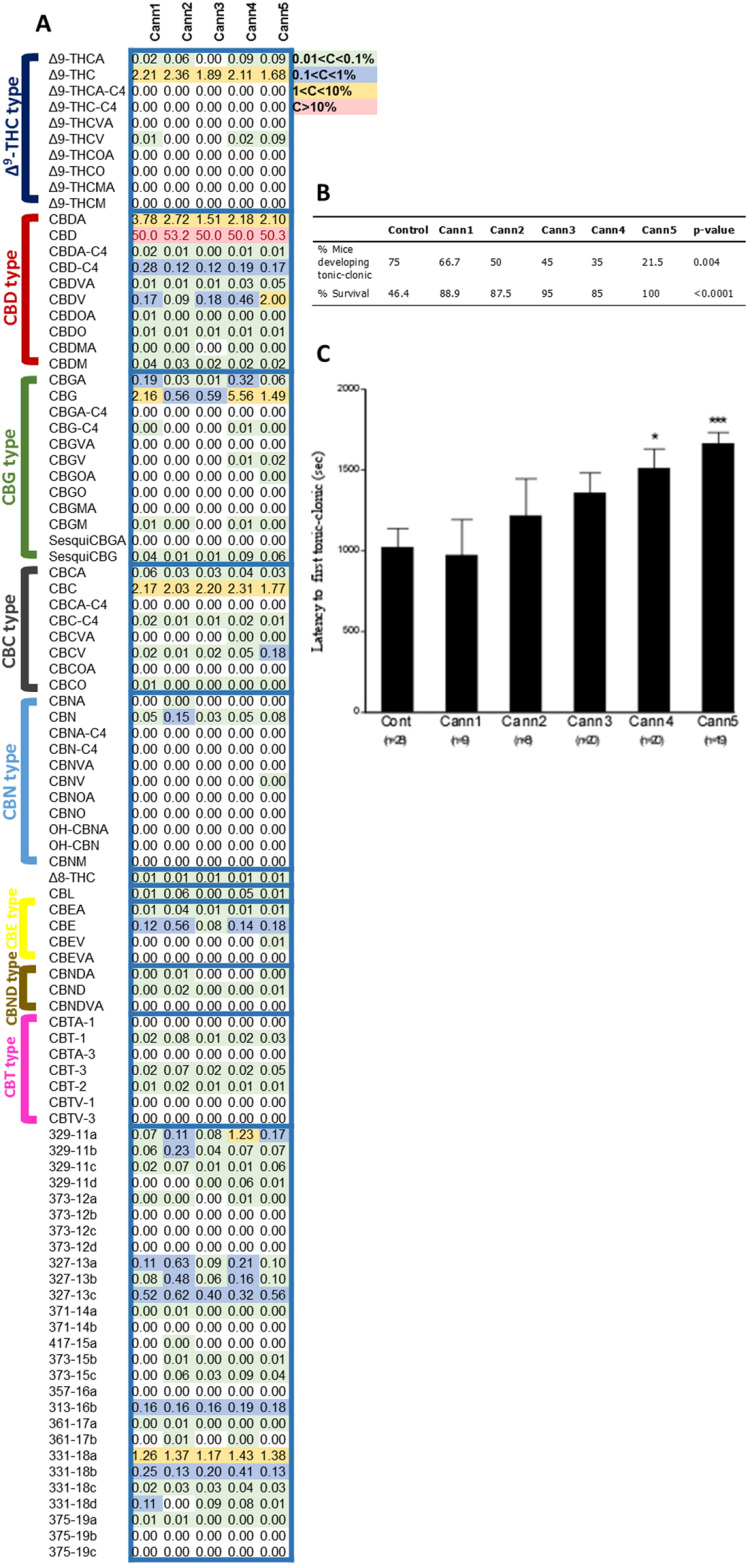
Effect of equally high CBD *Cannabis* strain extracts on pentylenetetrazol (PTZ)-induced convulsions. (**A**) Comparison of the LC/MS concentrations of the five *Cannabis* extracts that were used to evaluate the anticonvulsant effect of *Cannabis* extracts using the PTZ-induced convulsion test in mice. Values were color-coded according to order of magnitude of concentrations. All strains contained similar CBD contents (50% w/w) but differed in the concentrations and ratios of other phytocannabinoids. (**B**) The analyzed *Cannabis* extracts were found to reduce PTZ-induced incidence of tonic-clonic seizures and mortality. (**C**) Both Cann4 and Cann5 extracts significantly increased latency to first tonic-clonic seizures (F_5,103_ = 4.4, p = 0.001). Values are reported as mean ± SEM, *p < 0.05, ***p < 0.001 vs. a control group. Percent of protection against incidence of tonic seizures and subsequent death to PTZ were compared among groups using the χ^2^ test (p > 0.05 for both seizure protection and mortality protection).

In order to further demonstrate the importance of comprehensive phytocannabinoid analysis we chose to test whether equally high CBD *Cannabis* strains have different anticonvulsant properties. To this end, we used the pentylenetetrazol (PTZ) test in mice to evaluate the anticonvulsant properties of five different equally high CBD strains which differed by the content of other non-classical phytocannabinoids. The PTZ test is a validated model commonly used to identify drugs that are effective in the treatment of epilepsy in humans. As the levels of CBD slightly differed between the strains, we normalized each one to 50% CBD either by adding pure CBD or by diluting the extract (Fig. 6A). In panel 6A we also present the calculated absolute concentrations of all 94 cannabinoids for the five strains according to the suggested LC/MS method. After administration of PTZ, mice were observed and ranked according to a seizure profile by measuring latencies to seizures (clonic/tonic) and evaluating protection against incidence of tonic-clonic seizures and death subsequent to PTZ. Although, all high- CBD extracts were effective in reducing PTZ-induced death and reducing the incidence of tonic-clonic seizures, only Cann5 protected all mice tested and induced the lowest incidence of mice developing tonic-clonic seizures (Fig. 6B). In addition, Cann4 and Cann5 extracts were significantly effective in increasing the latency to first tonic-clonic seizures (Fig. 6C). These results suggest that not all high-CBD extracts have the same anticonvulsant properties, and that comprehensive phytocannabinoid profiling can enable to evaluate the potential anticonvulsant properties of *Cannabis* extracts.

## Discussion

*Cannabis*’s value and potential is changing all over the world. Patients, physicians, and governmental bodies are giving increased attention to medical *Cannabis*. In the past ten years, there has been a rapid growth in the discovery and use of *Cannabis*-based extracts for various therapeutic and medical purposes. The number of people worldwide that are currently using physician-prescribed medical *Cannabis* is estimated at a few millions^47^. According to the ProCon organization, in the U.S. alone, as of 2018, this number was over 2.1 million patients^47^.

To date, there have been over 500 different compounds reported to be found within the *Cannabis* plant^1^ of which at least 144 were classified as phytocannabinoids^19^. Recent findings suggest that different phytocannabinoids exhibit diverse pharmacological and biological activities and that they act on multiple targets. A recent review by Russo^48^ supports this supposition stating that phytocannabinoids and combinations of cannabinoids can, in certain situations, be more effective than Δ^9^-THC or CBD alone. In 2015, Dr. Sanchez-Ramos described how physicians may not be aware that prescribing Δ^9^-THC alone (e.g., Marinol^®^ or dronabinol) may not be as efficacious as utilizing the full plant for many types of patients suffering from cancer, neuropathic pain, multiple sclerosis, and bladder-diseases^49^. Turner *et al*.^17^ and Morales *et al*.^18^ suggested that the relative proportions of each phytocannabinoid type will additionally influence the pharmacological effects of whole *Cannabis* extracts, either through a polypharmacological effect of the phytocannabinoids themselves, or through modulation of phytocannabinoid effects by the non-cannabinoid content of the plant since they act on multiple targets.

It is therefore crucial to derive accurate and comprehensive measurements of *Cannabis* phytocannabinoid contents. In the presented work, we established an LC/MS/MS spectral mass library consisting of 94 phytocannabinoids from all 10 phytocannabinoid subclasses (Supplementary Figs S3–S13) with different levels of confidence as previously described. This developed MS/MS spectral library can be directly employed by other experts with access to similar instrumental configurations for putative identification of unknown peaks in *Cannabis* extracts, as generally performed using public/commercial spectral libraries. Moreover, one could develop in-house MS/MS spectral libraries following the chromatographic and MS characteristics suggested in this research for phytocannabinoid identification.

In terms of reliable quantification of absolute concentrations using LC/MS, analytical standards are needed since IEs of components with different chemical structures are difficult to predict. Herein lies the difficulty with phytocannabinoid quantification, as commercial standards for most phytocannabinoids found in the plant are not available. In the presented work, absolute concentrations for each phytocannabinoid in the 36 samples were calculated. However, absolute quantification in this case should be regarded with different levels of confidence. For type I phytocannabinoids, for which analytical standards were available, external calibration curves were developed for quantification. This method was validated by a comparison of concentrations for the same sample with the widely accepted standard addition method^50,51^. No statistical difference was observed for the two methods of quantification (p = 0.13). For all other phytocannabinoids, concentrations were extrapolated by comparing IEs of the type I phytocannabinoids (Supplementary Table S3), as described in the results chapter. According to the calibration curves of the largest and smallest IEs of the available analytical standards we estimated deviations from reported concentrations for each phytocannabinoid. Although large deviations can be observed as a result of extrapolation of calculated absolute concentrations for each phytocannabinoid, this method of quantification can be useful for identifying compounds that are more probable to produce biological effects based on their expression levels.

To understand variations in phytocannabinoid profiles for a large set of *Cannabis* samples a differential analysis was employed, by which samples were compared per phytocannabinoid. Analyzing the full spectrum of phytocannabinoids in the *Cannabis* plant is critical for understanding its therapeutic potential as each *Cannabis* strain contains a different chemical composition (Figs 3 and 4, and Supplementary Fig. S14A,B). Moreover, the maturation state, harvest, and growing conditions are also important factors which play a role and can alter the phytocannabinoid profile for a specific strain (Fig. 5A–I). Degradation or alteration of the biosynthesized phytocannabinoids can be dependent on storage time and methods used to process, extract and formulate the plant material. All these variable and unpredictable conditions may change a well-defined *Cannabis* preparation into a product with significantly different biological effects^17,52^.

An example of the complexity involved in identifying phytocannabinoid compositions for treatment purposes is given in Fig. 5A–I. As preparation for a clinical trial, one grower grew genetically identical *Cannabis* strains which were suspected to predominantly produce copious quantities of CBGVA. All the *Cannabis* plants were planted and harvested the same way but grown in four different greenhouses. Figure 5A revealed that genetically-identical *Cannabis* strains can express different phytocannabinoid compositions. This shows that even when choosing a specific strain, a patient may be given a medication whose phytocannabinoid profile will not produce the desired effect as phytocannabinoid types and ratios may differ widely.

The importance for full phytocannabinoid profiling was also demonstrated by the PTZ test in mice. This PTZ test was used to evaluate the anticonvulsant properties of 5 different equally high CBD extracts with varying contents of non-classical phytocannabinoids (Fig. 6A). Since epilepsy patients today get *Cannabis* extracts based almost exclusively on CBD levels, we felt that it was important to show that non-classical phytocannabinoids can affect the anticonvulsant properties of *Cannabis* and not to evaluate the anticonvulsant properties of specific strains. According to the presented data we found that despite the similarity in CBD contents, the *Cannabis* extracts produced different anticonvulsant effects, emphasizing the need to identify and quantify all other phytocannabinoids in these extracts (Fig. 6B,C). Furthermore, our results suggest that other non-classical phytocannabinoids can interfere with the anticonvulsant properties of CBD as some of the strains demonstrated lower anticonvulsant properties than others. Future work is needed to elucidate the roles and mechanisms that differing *Cannabis* components play in relation to potential anticonvulsant properties.

Crippa *et al*.^8^, recently described an improvement of symptoms in two cases of children with treatment-resistant epilepsy after they were administered a CBD-enriched extract (16% w/w CBD). In these two cases the initial administrations of the CBD-enriched extract eliminated seizures and improved general behavior, speech, understanding, and attention. In both cases, after three to four months of seizure reductions, symptoms reappeared and other side effects emerged. An analysis of the *Cannabis* extract (which they thought remained the same during the initial treatment) revealed changes in the phytocannabinoid ratios (CBD versus Δ^9^-THC). The authors suggested that the changes in Δ^9^-THC contents were responsible for the children’s symptom changes. However, they could not exclude the possibility that the symptom differences occurred due to other non-analyzed chemical substances in the *Cannabis* extracts. This study underlines the need to analyze all the compounds in an extract as it was not clear what was in the initial prescribed extract and what changed over time. This is the case with most of the data that has been collected today on medical *Cannabis*. Without identifying the whole extract content, this leads to a situation in which even when *Cannabis* treatments are reported as clinically useful one cannot effectively extrapolate insights from them and the treatments are seldom reproducible.

These case studies highlight the great need for accurate analysis of phytocannabinoid compositions before and throughout medical *Cannabis* clinical trials, treatments, or experiments. This is particularly important when prescribing *Cannabis* as a medication in terms of both therapeutic potential and side effects. Therefore, the exploration and identification of as many *Cannabis* components as possible is critical for exploiting the full potential of this unique plant and its derivatives.

The suggested approach in this research can be applied as a straightforward analytical method to robustly detect a wide range of phytocannabinoids in *Cannabis*, with a high potential for successful transfer into other laboratories. Our proposed method aims to advance the development and standardization of *Cannabis*-based medicines and to dramatically improve our fundamental understanding of how to determine optimal *Cannabis* treatments for specific patients.

## Methods

### Chemicals and reagents

LC/MS grade acetonitrile, methanol, and water for the mobile phase, and HPLC grade ethanol for sample preparation were obtained from Mercury Scientific and Industrial Products Ltd. (Rosh Haayin, Israel). LC/MS grade acetic acid was purchased from Sigma-Eldrich (Rehovot, Israel). The phytocannabinoid analytical standards (>98%) Δ^9^-THCA, Δ^9^-THC, Δ^8^-THC, CBN, CBC, CBD and CBG were purchased from Silicol Scientific Equipment Ltd. (Or Yehuda, Israel), CBDA and Δ^9^-THCV were purchased from Echo Pharmaceuticals BV (Weesp, The Netherlands), and CBGA, CBDV, CBDVA and CBL were purchased from Sigma-Eldrich (Rehovot, Israel).

### Phytocannabinoids extraction and sample preparation

Air-dried medical *Cannabis* female flowers grown in greenhouses were obtained from several local medical *Cannabis* distributors in sealed bags with 12–14% w/w moisture. We aimed to acquire and test the same types of products which were also distributed to patients. *Cannabis* flowers were manually crushed and 100 mg were accurately weighed and extracted with 10 ml ethanol. Samples were sonicated in an ultrasonic bath for 30 min and then agitated in an orbital shaker at 25 °C for 15 min. Samples were then allowed to settle at room temperature. A fraction of the supernatant was collected and filtered through a 0.22 µm PTFE syringe filter for analysis. For development of the LC/MS/MS phytocannabinoid library, samples were analyzed without further dilution. For quantification, samples were diluted in the ratios of 1:9, 1:99 and 1:999 v/v *Cannabis* extract to ethanol.

### Identification of phytocannabinoids by data dependent LC/MS/MS

Identification of phytocannabinoids was performed by analyzing the analytical phytocannabinoid standards and *Cannabis* samples in the prepared concentration. Data dependent MS/MS mode was performed using a Thermo Scientific UHPLC system coupled with a Q Exactive™ Hybrid Quadrupole-Orbitrap MS (Thermo Scientific, Bremen, Germany). The chromatographic separation was achieved using a Kinetex C18 core-shell column (2.6 μm, 150 mm × 2.1 mm i.d.) with a guard column (0.5 μm depth filter × 0.1 mm) (Phenomenex, Torrance, CA, USA) and a ternary A/B/C multistep gradient (solvent A: 0.1% acetic acid in Milli Q water, solvent B: 0.1% acetic acid in acetonitrile, and solvent C: methanol, all solvents were of LC/MS grade). Solvent C was kept constant at 5% throughout the run. The multistep gradient program was established as follows: initial conditions were 50% B raised to 67% B until 2 min, held at 67% B for 4 min, and then raised to 90% B until 10 min, held at 90% B until 14 min, decreased to 50% B over the next min, and held at 50% B until 20 min for re-equilibration of the system prior to the next injection. A flow rate of 0.3 ml/min was used, the column temperature was 30 °C and the injection volume was 1 μL. MS acquisition was carried out with a heated electro spray ionization (HESI-II) ion source operated in negative mode. Source parameters were as follows: sheath gas flow rate, auxiliary gas flow rate and sweep gas flow rate: 50, 20 and 0 arbitrary units respectively; capillary temperature: 350 °C; heater temperature: 50 °C; spray voltage: 3.00 kV. The scan range was 150–550 m/z for all acquisition events. MS was operated in full MS1 followed by data dependent MS/MS mode using the inclusion list in Supplementary Table S1, with an NCE of 40. Due to the time limit for scanning of masses at each MS/MS cycle, and considering the high variation and potential for overlapping of the analyzed masses, the list in *SI Appendix*, Table S1 was divided into two separate lists for the acid and neutral forms. Hence, tandem MS analyses were performed using separate lists for untreated and decarboxylated *Cannabis* extracts. Data acquisition in full MS1 mode was performed at 70,000 resolution, and the AGC target was set to 10^6^ with a maximum IT of 100 ms. Data acquisition in data dependent MS/MS mode was performed at 17,500 resolution, the AGC target was set to 10^5^ with a maximum IT of 50 ms and an isolation window of 4 m/z. To study the response of the major product ions of selected phytocannabinoids with energy, analytical standards and selected samples were analyzed in the range of 20–60 NCE levels.

### Phytocannabinoids profiling by LC/MS

For the differential profiling, the LC/MS instrument was operated in full MS1 mode by the same chromatographic and MS operational conditions and parameters used for the identification of the phytocannabinoids. Absolute quantification of phytocannabinoids with analytical standards was performed by external calibrations. Standard mixes were prepared ranging from 1 to 1000 ng/ml for Δ^9^-THCA, Δ^9^-THC, CBDA and CBD, and 0.25 to 625 ng/ml for all the other components. The dynamic range for each component was determined as maximum deviation from expected concentrations of 20%, and minimum signal-to-noise ratios of 10. Standard addition curves were performed by spiking one *Cannabis* extract with three different concentrations of standard compounds. In order to fit into the dynamic range of each component, the *Cannabis* sample was prepared in three dilutions (1:9, 1:99 and 1:999 v/v *Cannabis* extract to ethanol), and the analytical standards were added to the appropriate sample dilution.

Since no analytical standards were available for the additional phytocannabinoids identified in this research, linear dynamic ranges for each component were determined as follows: Two high-Δ^9^-THC and high-CBD *Cannabis* strains were chosen. A portion of the two samples was decarboxylated in an oven, and phytocannabinoids from the untreated and decarboxylated samples were extracted in ethanol. These four samples were then serially diluted in the ranges of 100 to 0.0001% v/v of the original concentrations. Each concentration was injected to the LC/MS in duplicates, and data was acquired in full MS1 mode. The dynamic range for each component was determined as maximum deviation from expected concentrations of 20%, and minimum signal-to-noise ratios of 10.

### Hierarchical cluster analysis

The hierarchical cluster analysis was executed in MATLAB R2016B (Mathworks, Nat-ick, MA, USA) on the normalized data according to maximum value of each phytocannabinoid. Data reordering for the heat map was performed according to an agglomerative hierarchical clustering algorithm. The distances between clusters were calculated according to Ward’s method for Euclidean metric. Analysis of results was carried out using the distance dendrogram.

### Study of the effect of equally high CBD *Cannabis* strain extracts on PTZ-induced convulsions

#### Mice

Adult (8–10 weeks of age; 20–25 g) male C57BL/6 mice (C57BL/6 J; The Jackson Laboratory) were used in all experiments. Mice were maintained under specific-pathogen-free (SPF) conditions and five mice were housed in each cage on a 12:12-h light cycle (lights on at 07:00). All experiments were performed in accordance with the National Institutes of Health’s *Guide for the Care and Use of Laboratory Animals*. All procedures and protocols were approved by the Technion Administrative Panel of Laboratory Animal Care (#:IL_130-11-2015).

#### Cannabis extraction and administration

Air dried medical *Cannabis* strains obtained from several local medical *Cannabis* distributors were decarboxylated and then extracted in ethanol as previously described. Following extraction ethanol was evaporated under reduced pressure at 38 °C using a rotary evaporator (Laborata 4000; Heidolph Instruments GmbH & Co. KG; Germany). The extracts were reconstituted into a vehicle solution consisting of 1:1:18 ethanol:cremophor (Sigma–Aldrich):saline to a final concentration of 20 mg/ml. CBD content for all the extracts was found to be in the range of 46.6 to 64.2% (w/w). To achieve equal contents of CBD for all the extracts we normalized each one to approximately 50% CBD either by adding pure CBD (>99%) or by diluting the extract by the vehicle solution. The *Cannabis* extract was injected intraperitoneally (150 mg/kg) 30 min before PTZ injections similarly to Jones *et al*.^53^ and Klein *et al*.^54^. A sample of the extract before formulation was analyzed using LC/MS by the described method for phytocannabinoid profiling.

Following *Cannabis* or control injections mice were subcutaneously injected with PTZ (80 mg/kg) and then monitored for 30 min. The epileptic seizures were captured using a video recording epileptic behavioral analysis system (SeizureScan, Clever Sys., Inc., Virgina, USA). During this time, the progression of seizure activity from normal behavior (P0) to behavior arrest (P1), to twitches (P2), to forelimb clonus (P3), to generalized clonic seizures (P4), to jumpy/bouncy seizures (P5) was analyzed.

### Statistical Analyses

For comparison of outcomes of the external calibration and standard addition method, assuming normally distributed experimental error, we performed *z*-score transformation for standard addition results using mean and standard deviation values learned from the corresponding external calibration measurements, for each compound and concentration separately. To determine the probability of having the experimental error of standard addition indistinguishable from the experimental error of the external calibration approach, sum of squared *z*-scores was compared to the cumulative distribution function of χ^2^ distribution with the degree of freedom equal to the number of summands. Determined probability was reported as a p-value for the test.

One-way ANOVA was used to determine statistical significance of four samples from the same high-CBDA strain grown in different greenhouses. P-values were corrected for multiple testing using the Tukey honest significant difference (HSD) post hoc test (*p < 0.05, **p < 0.01, ***p < 0.001, ****p < 0.0001).

In the study of PTZ-induced convulsions on mice latency to first clonic-tonic data were expressed as mean values ± SEM. Statistical analysis was performed using JMP 12 (SAS Institute Inc., 2015) software. Latency to first PTZ-induced generalized tonic-clonic seizure with loss of writing reflex was compared between experimental groups using one-way analysis of variance followed by comparisons with control using Dunnett’s methods post hoc test. P-values less than 0.05 were considered to be statistically significant. Percent of protection against incidence of tonic-clonic seizures and death subsequent PTZ was compared among groups using the χ^2^ test (p > 0.05 for both seizure protection and mortality protection).

## Electronic supplementary material

Supplementary Information
