# Supplementary material for: A new ESI-LC/MS approach for comprehensive metabolic profiling of phytocannabinoids in Cannabis
Source: Sci Rep. 2018 Sep 24;8:14280. doi: 10.1038/s41598-018-32651-4 (PMC6155167; doi:10.1038/s41598-018-32651-4)
Supplement: Supplementary file 1 — Supplementary Information [file 41598_2018_32651_MOESM1_ESM.pdf]

Supporting Information for Publication

A new ESI-LC/MS approach for comprehensive metabolic profiling of phytocannabinoids in *Cannabis*

Paula Berman[a], *Kate Futoran*[a], *Gil M Lewitus*[a], *Dzmitry Mukha*[a], *Maya Benami*[a], *Tomer Shlomi*[a,b], *and David Meiri*[a,*]

[a]Department of Biology, Technion-Israel Institute of Technology, Haifa 320003, Israel; [b]Department of Computer Science, Technion-Israel Institute of Technology, Haifa 320003, Israel.

[*]Corresponding author: Tel/fax: +972-77-8871680; Email: dmeiri@technion.ac.il

Table of contents

List of Supplementary Figures

Figure S1. General overview of work methodology.......... S-1
Figure S2. Plots of the LC/MS/MS ion abundance of the deprotonated molecular and product ions as a function of NCE energy for the available neutral phytocannabinoid standards. S-2
Figure S3. MS/MS spectral library of CBG-type phytocannabinoids.......... S-3
Figure S4. MS/MS spectral library of Δ^9-THC-type phytocannabinoids.......... S-4
Figure S5. MS/MS spectral library of CBD-type phytocannabinoids.......... S-5
Figure S6. MS/MS spectral library of CBC-type phytocannabinoids.......... S-6
Figure S7. MS/MS spectral library of CBN-type phytocannabinoids.......... S-7
Figure S8. MS/MS spectral library of Δ^8-THC phytocannabinoid.......... S-8
Figure S9. MS/MS spectral library of CBL phytocannabinoid.......... S-9
Figure S10. MS/MS spectral library of CBND-type phytocannabinoids.......... S-10
Figure S11. MS/MS spectral library of CBE-type phytocannabinoids.......... S-11
Figure S12. MS/MS spectral library of CBT-type phytocannabinoids.......... S-12
Figure S13. MS/MS spectral library of additional phytocannabinoids (Parts I-IV).......... S-13-S-16
Figure S14. Data analysis methods for exploring variations of phytocannabinoids for the 36 *Cannabis* samples.......... S-17

List of Supplementary Tables

Table S1. List of neutral and acid potential masses of phytocannabinoids used in data dependent MS/MS mode.......... S-18-S-23
Table S2. Validation of the external calibration curves for the available 13 phytocannabinoids.......... S-24
Table S3. Ranges of absolute phytocannabinoid contents for the 36 *Cannabis* samples.......... S-25-S-28

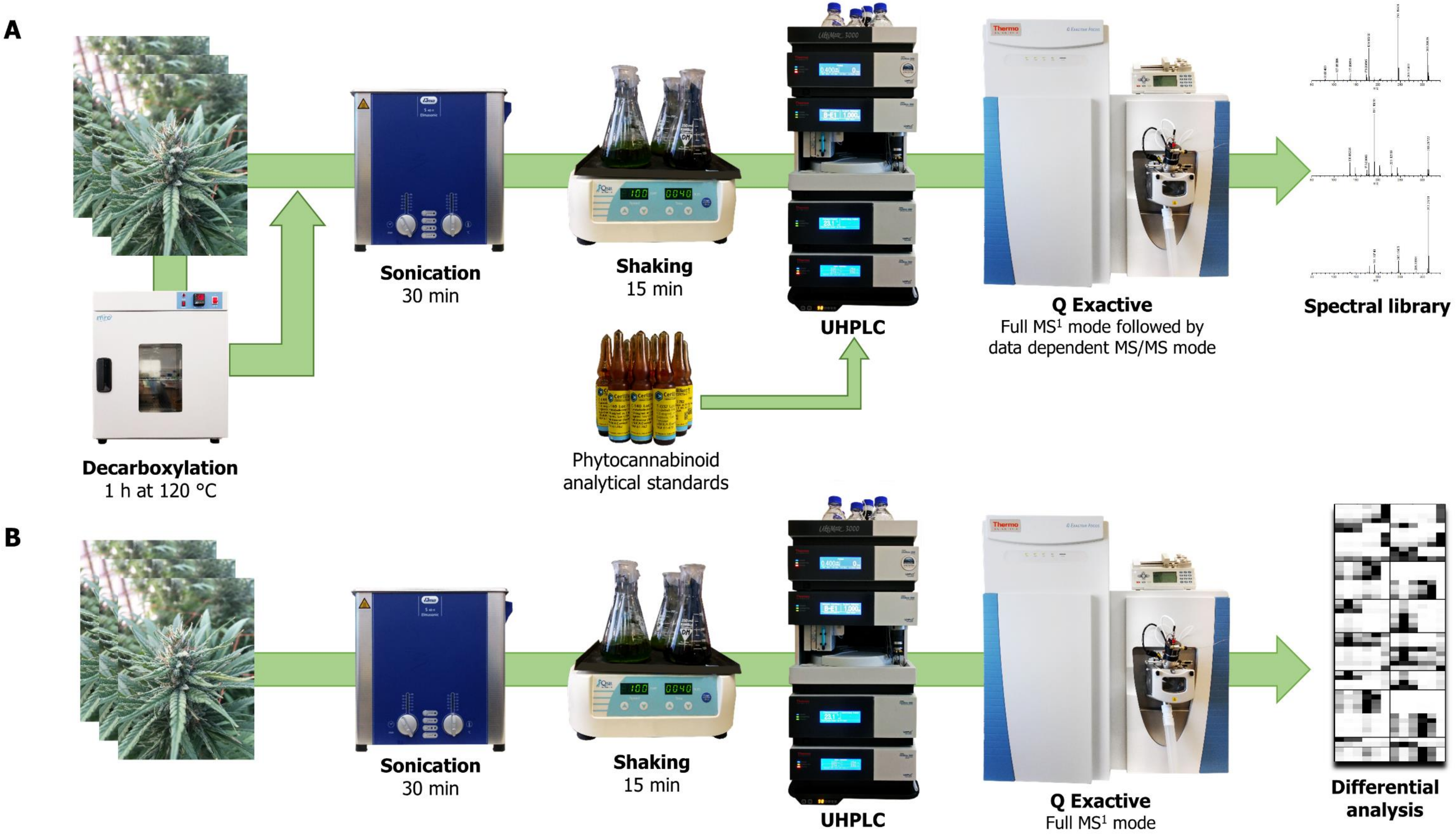

Figure S1. General overview of work methodology. (**A**) In the identification process, phytocannabinoids were chromatographically separated by reversed phase ultra HPLC (RP-UHPLC) and detected via MS/MS analysis. (**B**) Differential profiling of numerous medical *Cannabis* strains was performed by LC/MS according to the retention times and accurate masses of the identified phytocannabinoids in the spectral MS/MS library.

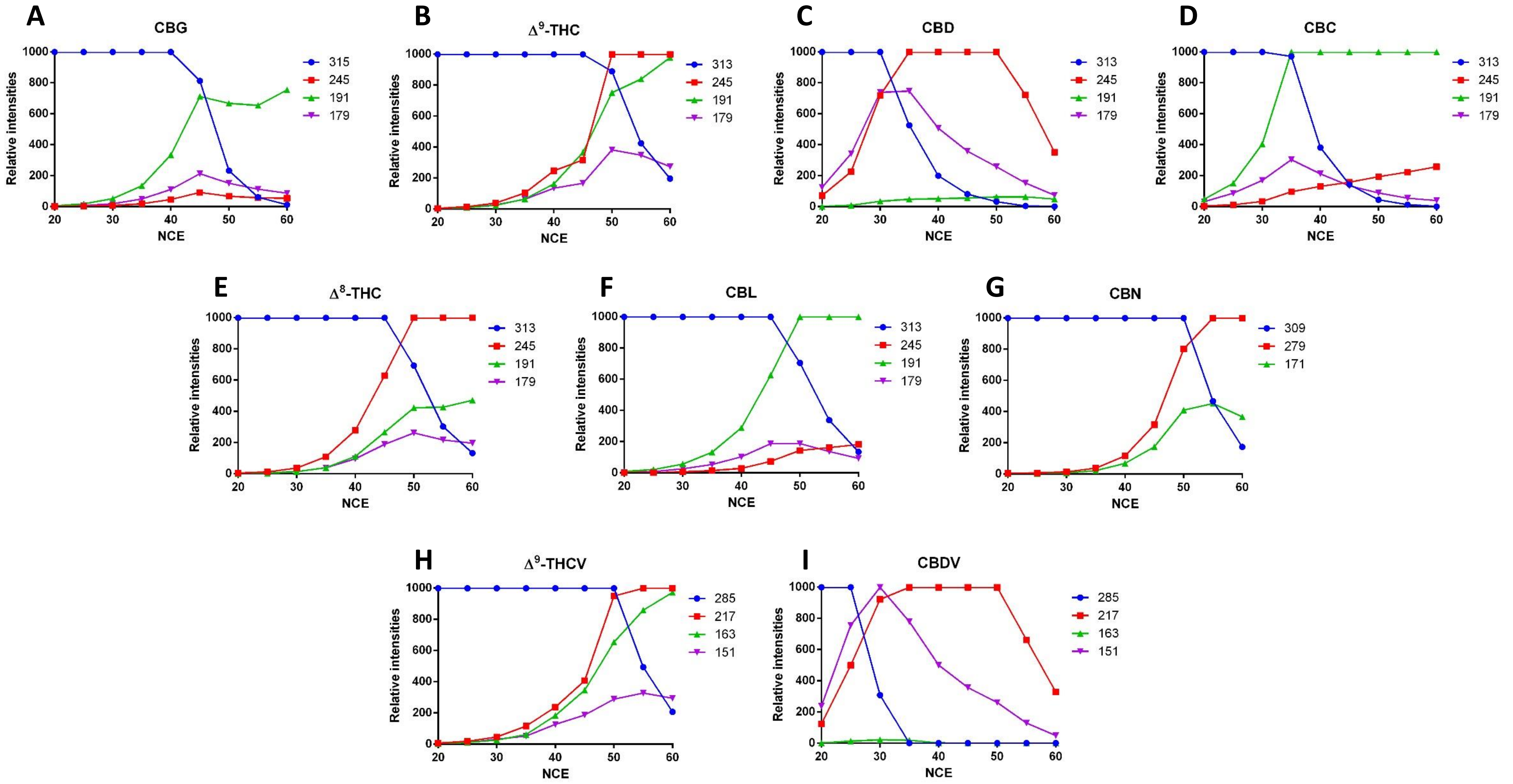

Figure S2. Plots of the LC/MS/MS ion abundance of the deprotonated precursor and product ions as a function of NCE energy for the available neutral pentyl and propyl phytocannabinoid standards. (**A**) CBG, (**B**) Δ^9-THC, (**C**) CBD, (**D**) CBC, (**E**) Δ^8-THC, (**F**) CBL, (**G**) CBN, (**H**) Δ^9-THCV, and (**I**) CBDV. Accurate masses according to high-resolution LC/MS in descending order are as follows: 315.2329, 313.2173, 309.1860, 285.1860, 279.1391, 245.1547, 217.1234, 191.1078, 179.1078, 171.0815, 163.0765 and 151.0765.

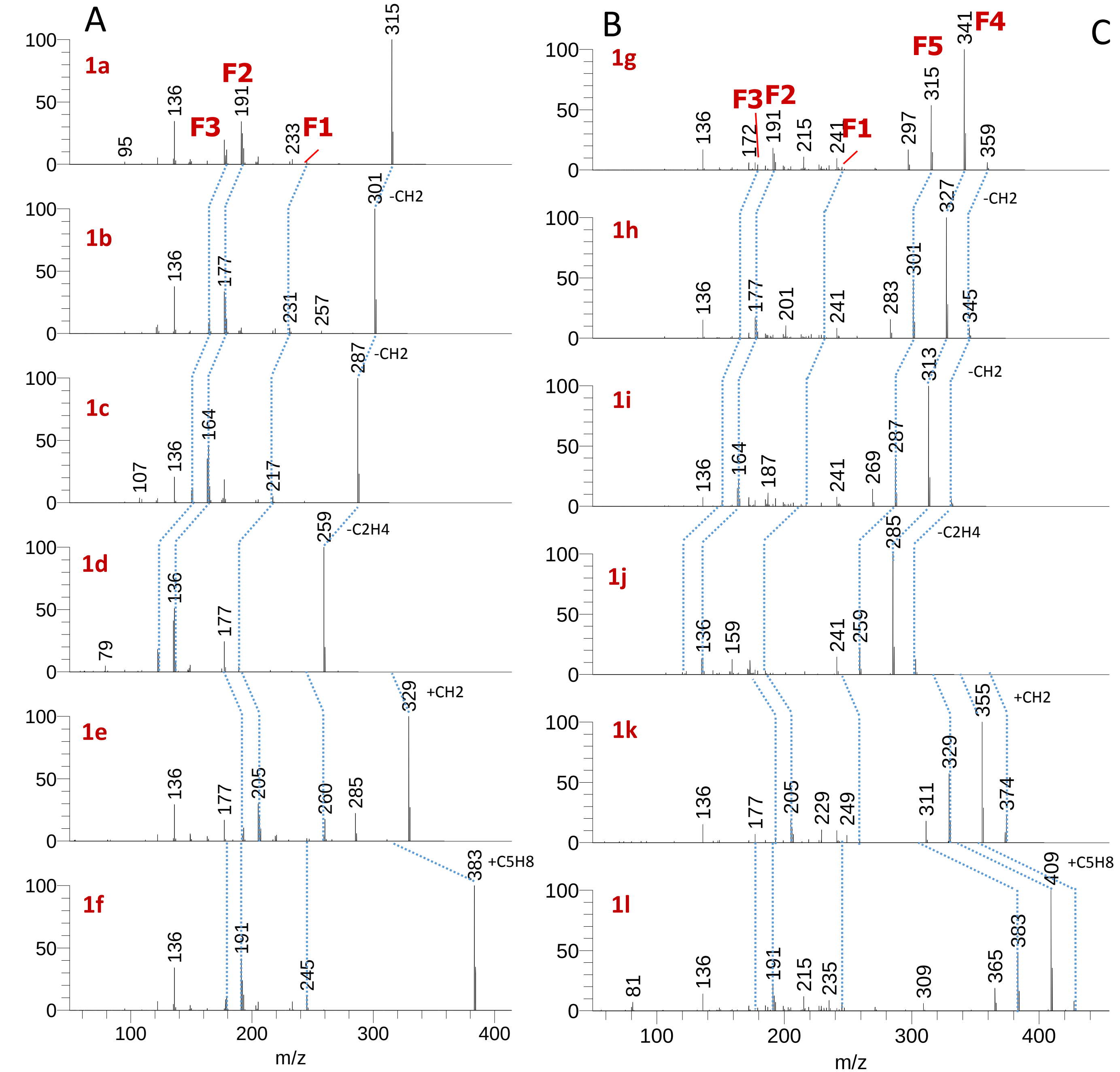

C

	Name	R1	R2	R3	R4	RT [min]	[M-H]-	[MF1-H]-	[MF2-H]-	[MF3-H]-	[MF4-H]-	[MF5-H]-
1a	CBG[a]	C5H11	H	H	H	7.86	315.2329	245.1547	191.1078	179.1067	-	-
							C21H31O2	C16H21O2	C12H15O2	C11H15O2		
1b	CBG-C4[b]	C4H9	H	H	H	6.70	301.2173	231.1391	177.0921	165.0921	-	-
							C20H29O2	C15H19O2	C11H13O2	C10H13O2		
1c	CBGV	C3H7	H	H	H	5.93	287.2017	217.1234	163.0765	151.0765	-	-
							C19H27O2	C14H17O2	C10H11O2	C9H11O2		
1d	CBGO[b]	CH3	H	H	H	4.82	259.1704	189.0921	135.0452	123.0452	-	-
							C17H23O2	C12H13O2	C8H7O2	C7H7O2		
1e	CBGM	C5H11	H	CH3	H	9.33	329.2486	259.1704	205.1234	193.1234	-	-
							C22H33O2	C17H23O2	C13H17O2	C12H17O2		
1f	SesquiCBG	C5H11	H	H	C5H9	12.17	383.2955	245.1547	191.1078	179.1067	-	-
							C26H39O2	C16H21O2	C12H15O2	C11H15O2		
1g	CBGA[a]	C5H11	COOH	H	H	8.21	359.2228	245.1547	191.1078	179.1067	341.2122	315.2329
							C22H31O4	C16H21O2	C12H15O2	C11H15O2	C22H29O3	C21H31O2
1h	CBGA-C4[b]	C4H9	COOH	H	H	7.15	345.2071	231.1391	177.0921	165.0921	327.1966	301.2173
							C21H29O4	C15H19O2	C11H13O2	C10H13O2	C21H27O3	C20H29O2
1i	CBGVA	C3H7	COOH	H	H	6.34	331.1915	217.1234	163.0765	151.0765	313.1809	287.2017
							C20H27O4	C14H17O2	C10H11O2	C9H11O2	C20H25O3	C19H27O2
1j	CBGOA[b]	CH3	COOH	H	H	5.37	303.1602	189.0921	135.0452	123.0452	285.1496	259.1704
							C18H23O4	C12H13O2	C8H7O2	C7H7O2	C18H21O3	C17H23O2
1k	CBGMA	C5H11	COOH	CH3	H	9.70	373.2384	259.1704	205.1234	193.1234	355.2279	329.2486
							C23H33O4	C17H23O2	C13H17O2	C12H17O2	C23H31O3	C22H33O2
1l	SesquiCBGA[b]	C5H11	COOH	H	C5H9	12.40	427.2854	245.1547	191.1078	179.1067	409.2748	383.2955
							C27H39O4	C16H21O2	C12H15O2	C11H15O2	C27H37O3	C26H39O2

[a]Analytical standard

[b]Non-isolated and identified phytocannabinoid in the literature

D

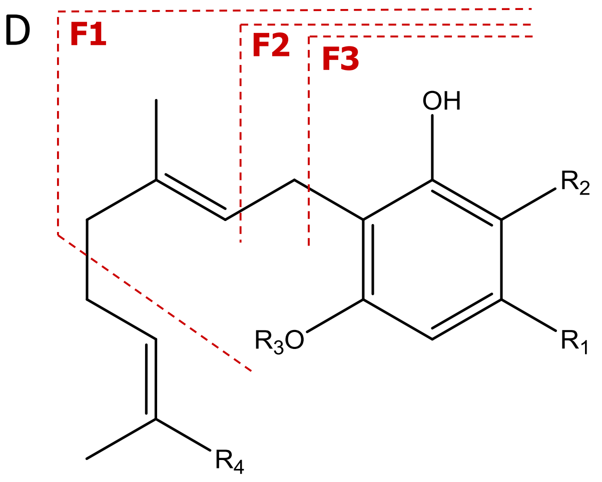

Figure S3. MS/MS spectral library of CBG-type phytocannabinoids. MS/MS spectra of the identified CBG type (**A**) neutral and (**B**) acid phytocannabinoids, (**C**) names, retention times and accurate masses, and (**D**) their fragmentation structures. Values of m/z in (**A**) and (**B**) are presented as nominal masses to improve interpretation of spectra. Accurate masses for the main fragments appear in (**C**).

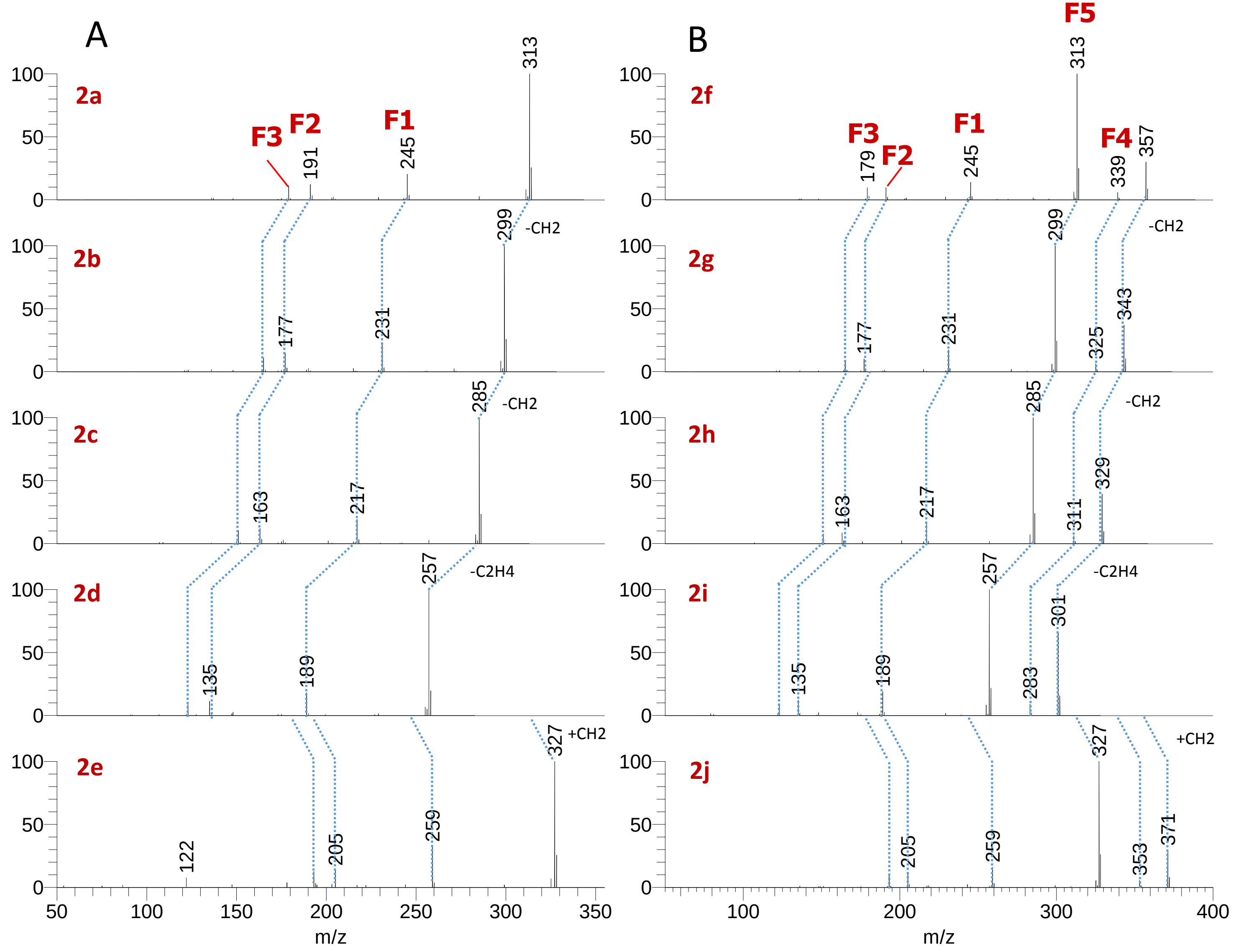

C

	Name	R1	R2	R3	RT [min]	[M-H]-	[MF1-H]-	[MF2-H]-	[MF3-H]-	[MF4-H]-	[MF5-H]-
2a	d9-THC[a]	C5H11	H	H	11.26	313.2173<br>$C_{21}H_{29}O_2$	245.1547<br>$C_{16}H_{21}O_2$	191.1078<br>$C_{12}H_{15}O_2$	179.1067<br>$C_{11}H_{15}O_2$	-	-
2b	d9-THC-C4	C4H9	H	H	9.92	299.2017<br>$C_{20}H_{27}O_2$	231.1391<br>$C_{15}H_{19}O_2$	177.0921<br>$C_{11}H_{13}O_2$	165.0921<br>$C_{10}H_{13}O_2$	-	-
2c	d9-THCV[a]	C3H7	H	H	8.16	285.1860<br>$C_{19}H_{25}O_2$	217.1234<br>$C_{14}H_{17}O_2$	163.0765<br>$C_{10}H_{11}O_2$	151.0765<br>$C_9H_{11}O_2$	-	-
2d	d9-THCO	CH3	H	H	5.86	257.1547<br>$C_{17}H_{21}O_2$	189.0921<br>$C_{12}H_{13}O_2$	135.0452<br>$C_8H_7O_2$	123.0452<br>$C_7H_7O_2$	-	-
2e	d9-THCM[b]	C5H11	H	CH3	12.32	327.2329<br>$C_{22}H_{31}O_2$	259.1704<br>$C_{17}H_{23}O_2$	205.1234<br>$C_{13}H_{17}O_2$	193.1234<br>$C_{12}H_{17}O_2$	-	-
2f	d9-THCA[a]	C5H11	COOH	H	12.71	357.2071<br>$C_{22}H_{29}O_4$	245.1547<br>$C_{16}H_{21}O_2$	191.1078<br>$C_{12}H_{15}O_2$	179.1067<br>$C_{11}H_{15}O_2$	339.1966<br>$C_{22}H_{27}O_3$	313.2173<br>$C_{21}H_{29}O_2$
2g	d9-THCA-C4	C4H9	COOH	H	11.76	343.1915<br>$C_{21}H_{27}O_4$	231.1391<br>$C_{15}H_{19}O_2$	177.0921<br>$C_{11}H_{13}O_2$	165.0921<br>$C_{10}H_{13}O_2$	325.1809<br>$C_{21}H_{25}O_3$	299.2017<br>$C_{20}H_{27}O_2$
2h	d9-THCVA	C3H7	COOH	H	10.52	329.1758<br>$C_{20}H_{25}O_4$	217.1234<br>$C_{14}H_{17}O_2$	163.0765<br>$C_{10}H_{11}O_2$	151.0765<br>$C_9H_{11}O_2$	311.1653<br>$C_{20}H_{23}O_3$	285.1860<br>$C_{19}H_{25}O_2$
2i	d9-THCOA	CH3	COOH	H	7.83	301.1445<br>$C_{18}H_{21}O_4$	189.0921<br>$C_{12}H_{13}O_2$	135.0452<br>$C_8H_7O_2$	123.0452<br>$C_7H_7O_2$	283.1340<br>$C_{18}H_{19}O_3$	257.1547<br>$C_{17}H_{21}O_2$
2j	d9-THCMA[b]	C5H11	COOH	CH3	13.54	371.2228<br>$C_{23}H_{31}O_4$	259.1704<br>$C_{17}H_{23}O_2$	205.1234<br>$C_{13}H_{17}O_2$	193.1234<br>$C_{12}H_{17}O_2$	353.2122<br>$C_{23}H_{29}O_3$	327.2329<br>$C_{22}H_{31}O_2$

[a]Analytical standard

[b]Non-isolated and identified phytocannabinoid in the literature

D

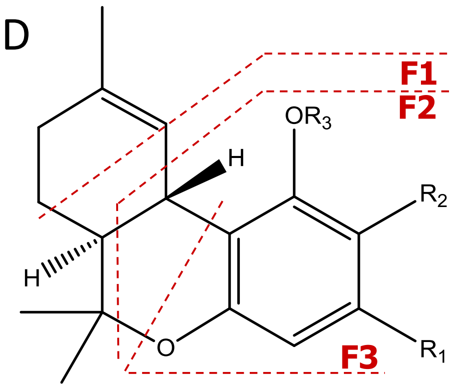

Figure S4. MS/MS spectral library of Δ^9-THC-type phytocannabinoids. MS/MS spectra of the identified Δ^9-THC type (**A**) neutral and (**B**) acid phytocannabinoids, (**C**) names, retention times and accurate masses, and (**D**) their fragmentation structures. Values of m/z in (**A**) and (**B**) are presented as nominal masses to improve interpretation of spectra. Accurate masses for the main fragments appear in (**C**).

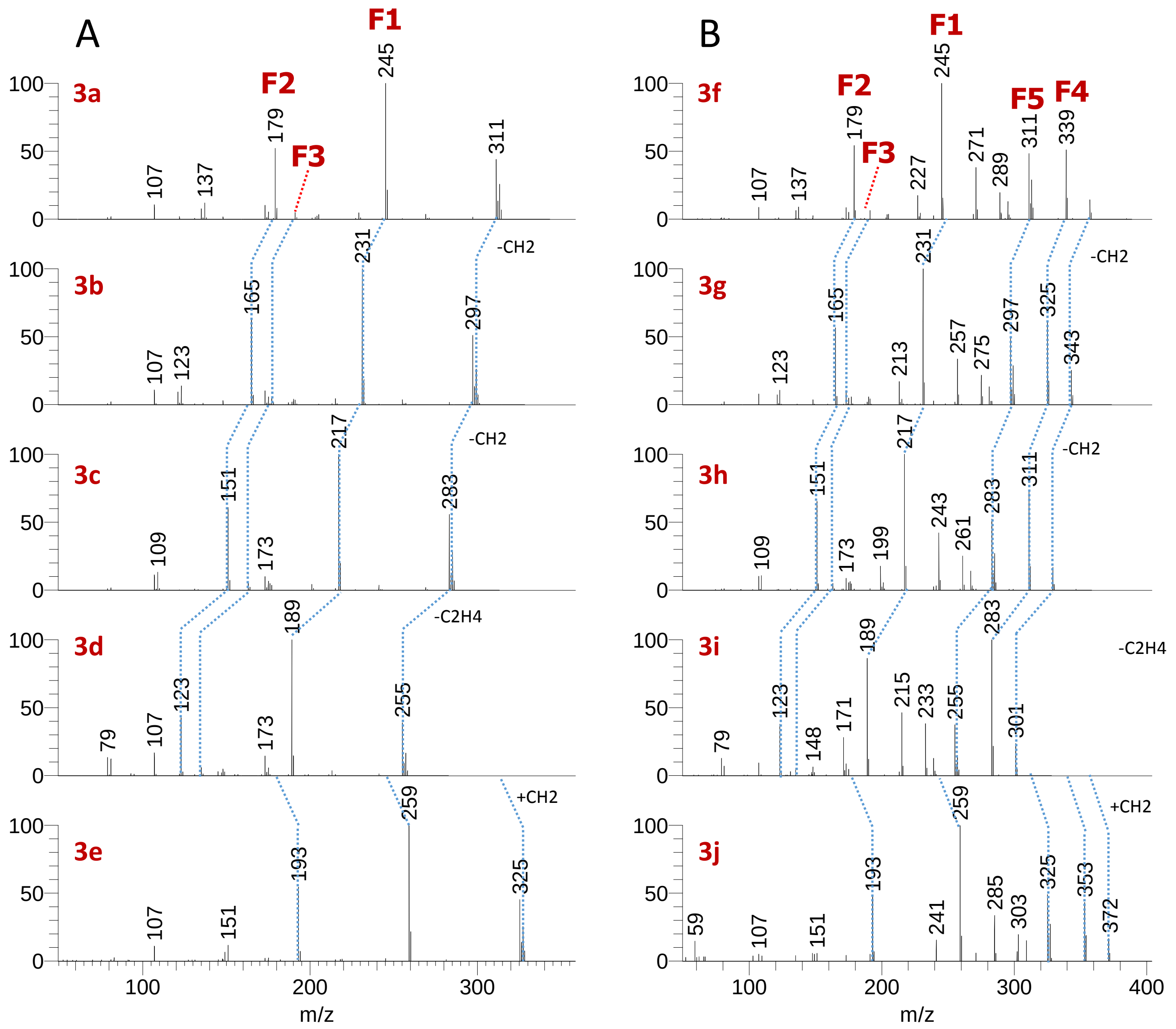

C

	Name	R1	R2	R3	RT [min]	[M-H]-	[MF1-H]-	[MF2-H]-	[MF3-H]-	[MF4-H]-	[MF5-H]-
**3a**	CBD[a]	C5H11	H	H	7.96	313.2173<br>C21H29O2	245.1547<br>C16H21O2	191.1078<br>C12H15O2	179.1067<br>C11H15O2	-	-
**3b**	CBD-C4	C4H9	H	H	6.74	299.2017<br>C20H27O2	231.1391<br>C15H19O2	177.0921<br>C11H13O2	165.0921<br>C10H13O2	-	-
**3c**	CBDV[a]	C3H7	H	H	5.86	285.1860<br>C19H25O2	217.1234<br>C14H17O2	163.0765<br>C10H11O2	151.0765<br>C9H11O2	-	-
**3d**	CBDO	CH3	H	H	4.61	257.1547<br>C17H21O2	189.0921<br>C12H13O2	135.0452<br>C8H7O2	123.0452<br>C7H7O2	-	-
**3e**	CBDM	C5H11	H	CH3	10.7	327.2329<br>C22H31O2	259.1704<br>C17H23O2	205.1234<br>C13H17O2	193.1234<br>C12H17O2	-	-
**3f**	CBDA[a]	C5H11	COOH	H	7.67	357.2071<br>C22H29O4	245.1547<br>C16H21O2	191.1078<br>C12H15O2	179.1067<br>C11H15O2	339.1966<br>C22H27O3	313.2173<br>C21H29O2
**3g**	CBDA-C4[b]	C4H9	COOH	H	6.64	343.1915<br>C21H27O4	231.1391<br>C15H19O2	177.0921<br>C11H13O2	165.0921<br>C10H13O2	325.1809<br>C21H25O3	299.2017<br>C20H27O2
**3h**	CBDVA[a]	C3H7	COOH	H	5.90	329.1758<br>C20H25O4	217.1234<br>C14H17O2	163.0765<br>C10H11O2	151.0765<br>C9H11O2	311.1653<br>C20H23O3	285.1860<br>C19H25O2
**3i**	CBDOA[b]	CH3	COOH	H	4.92	301.1445<br>C18H21O4	189.0921<br>C12H13O2	135.0452<br>C8H7O2	123.0452<br>C7H7O2	283.1340<br>C18H19O3	257.1547<br>C17H21O2
**3j**	CBDMA[b]	C5H11	COOH	CH3	9.16	371.2228<br>C23H31O4	259.1704<br>C17H23O2	205.1234<br>C13H17O2	193.1234<br>C12H17O2	353.2122<br>C23H29O3	327.2329<br>C22H31O2

[a]Analytical standard
[b]Non-isolated and identified phytocannabinoid in the literature

D

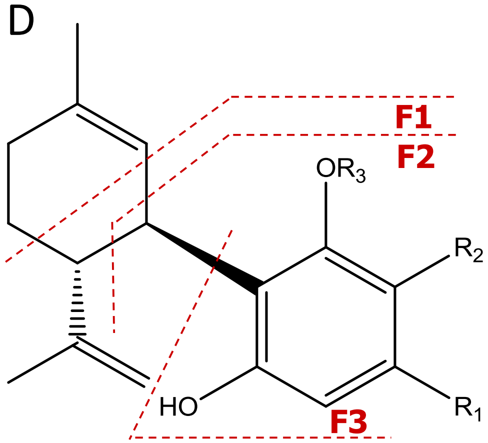

Figure S5. MS/MS spectral library of CBD-type phytocannabinoids. MS/MS spectra of the identified CBD type (**A**) neutral and (**B**) acid phytocannabinoids, (**C**) names, retention times and accurate masses, and (**D**) their fragmentation structures. Values of m/z in (**A**) and (**B**) are presented as nominal masses to improve interpretation of spectra. Accurate masses for the main fragments appear in (**C**).

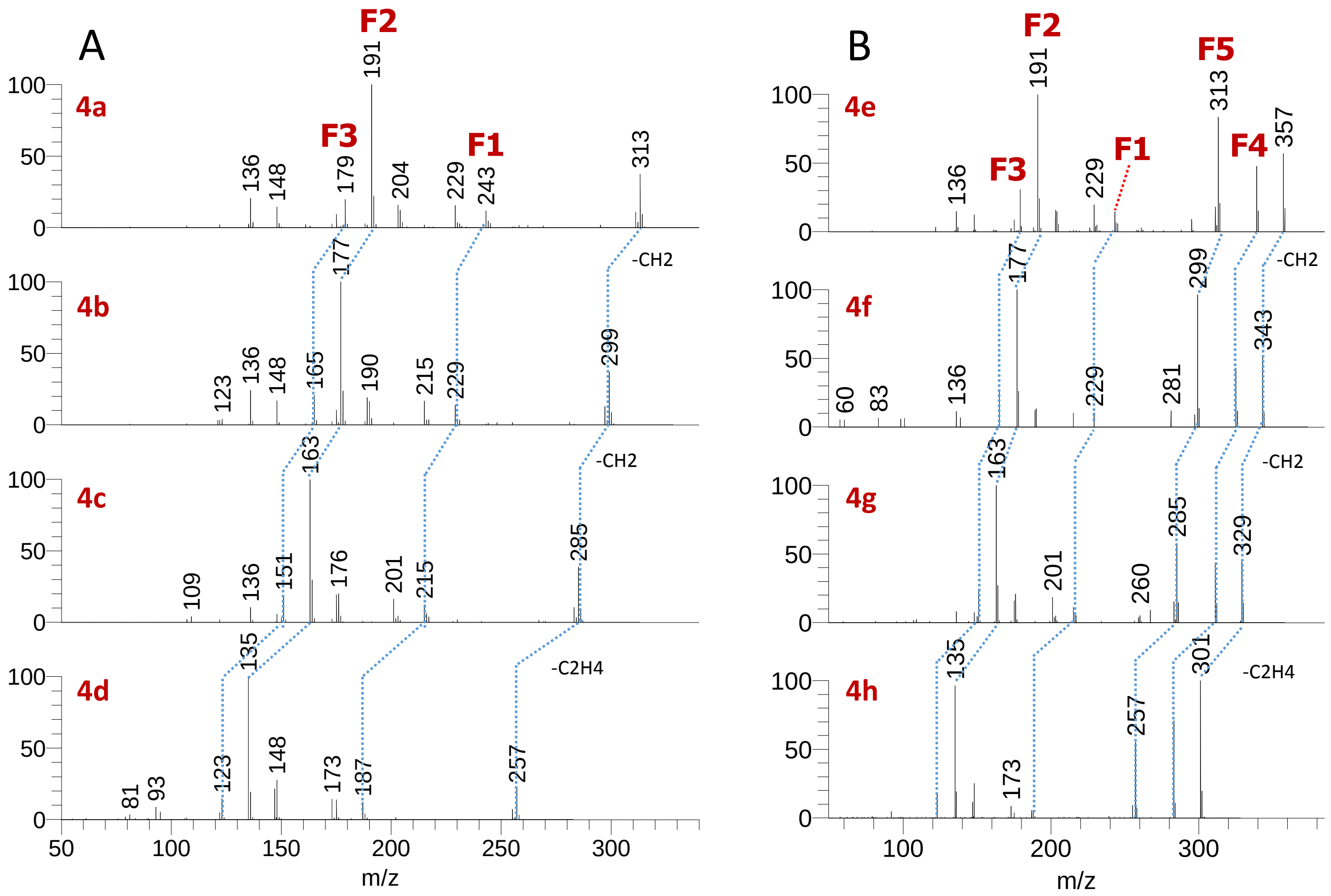

C

	Name	R1	R2	RT [min]	[M-H]-	[MF1-H]-	[MF2-H]-	[MF3-H]-	[MF4-H]-	[MF5-H]-
**4a**	CBC[a]	C5H11	H	12.08	313.2173 C21H29O2	243.1391 C16H19O2	191.1078 C12H15O2	179.1067 C11H15O2	-	-
**4b**	CBC-C4[b]	C4H9	H	11.05	299.2017 C20H27O2	229.1234 C15H17O2	177.0921 C11H13O2	165.0921 C10H13O2	-	-
**4c**	CBCV	C3H7	H	9.64	285.1860 C19H25O2	215.1078 C14H15O2	163.0765 C10H11O2	151.0765 C9H11O2	-	-
**4d**	CBCO	CH3	H	6.82	257.1547 C17H21O2	187.0765 C12H11O2	135.0452 C8H7O2	123.0452 C7H7O2	-	-
**4e**	CBCA	C5H11	COOH	13.23	357.2071 C22H29O4	243.1391 C16H19O2	191.1078 C12H15O2	179.1067 C11H15O2	339.1966 C22H27O3	313.2173 C21H29O2
**4f**	CBCA-C4[b]	C4H9	COOH	12.43	343.1915 C21H27O4	229.1234 C15H17O2	177.0921 C11H13O2	165.0921 C10H13O2	325.1809 C21H25O3	299.2017 C20H27O2
**4g**	CBCVA	C3H7	COOH	11.48	329.1758 C20H25O4	215.1078 C14H15O2	163.0765 C10H11O2	151.0765 C9H11O2	311.1653 C20H23O3	285.1860 C19H25O2
**4h**	CBCOA	CH3	COOH	9.36	301.1445 C18H21O4	187.0765 C12H11O2	135.0452 C8H7O2	123.0452 C7H7O2	283.1340 C18H19O3	257.1547 C17H21O2

[a] Analytical standard
[b] Non-isolated and identified phytocannabinoid in the literature

D

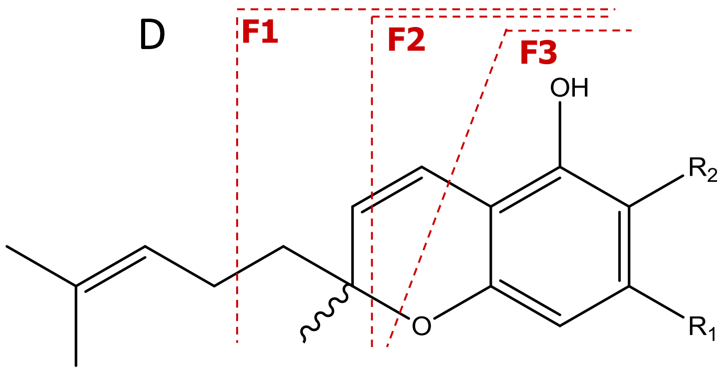

Figure S6. MS/MS spectral library of CBC-type phytocannabinoids. MS/MS spectra of the identified CBC type (**A**) neutral and (**B**) acid phytocannabinoids, (**C**) names, retention times and accurate masses, and (**D**) their fragmentation structures. Values of m/z in (**A**) and (**B**) are presented as nominal masses to improve interpretation of spectra. Accurate masses for the main fragments appear in (**C**).

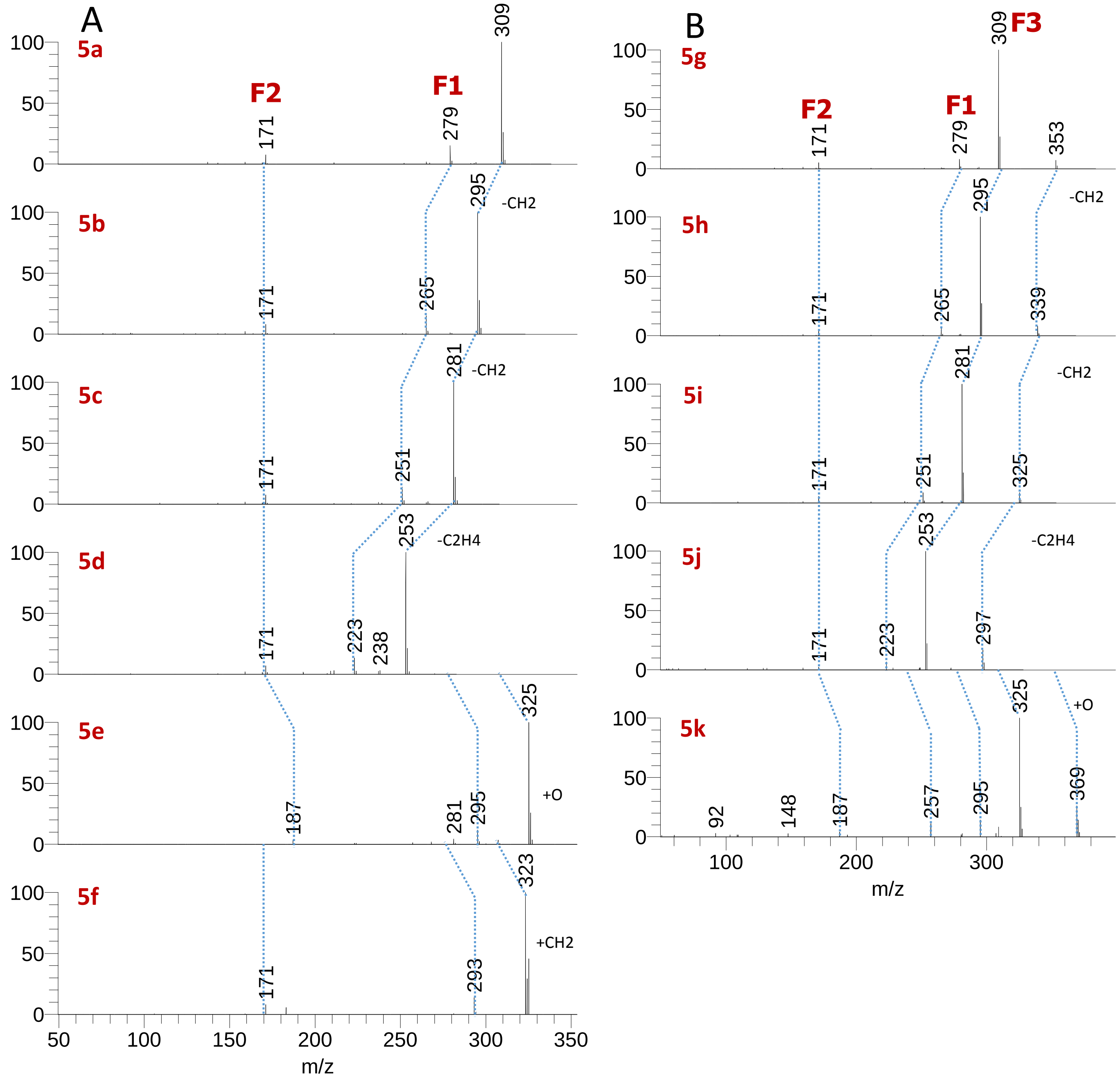

C

	Name	R1	R2	R3	R4	RT [min]	[M-H]-	[MF1-H]-	[MF2-H]-	[MF3-H]-
5a	CBN[a]	C5H11	H	H	H	10.3	309.1860 C21H25O2	279.1391 C19H19O2	171.0815 C12H11O	-
5b	CBN-C4	C4H9	H	H	H	8.57	295.1703 C20H23O2	265.1234 C18H17O2	171.0815 C12H11O	-
5c	CBNV	C3H7	H	H	H	7.08	281.1547 C19H21O2	251.1078 C17H15O2	171.0815 C12H11O	-
5d	CBNO	CH3	H	H	H	5.20	253.1234 C17H17O2	223.0765 C15H11O2	171.0815 C12H11O	-
5e	8-OH-CBN	C5H11	H	H	OH	9.34	325.1809 C21H25O3	295.1340 C19H19O3	187.0765 C12H11O2	-
5f	CBNM	C5H11	H	CH3	H	11.55	323.2016 C21H27O2	293.1547 C20H21O2	171.0815 C12H11O	-
5g	CBNA	C5H11	COOH	H	H	12.11	353.1758 C22H25O4	279.1391 C19H19O2	171.0815 C12H11O	309.1860 C21H25O2
5h	CBNA-C4[b]	C4H9	COOH	H	H	11.03	339.1602 C21H23O4	265.1234 C18H17O2	171.0815 C12H11O	295.1703 C20H23O2
5i	CBNVA[b]	C3H7	COOH	H	H	9.62	325.1445 C20H21O4	251.1078 C17H15O2	171.0815 C12H11O	281.1547 C19H21O2
5j	CBNOA[b]	CH3	COOH	H	H	7.14	297.1132 C18H17O4	223.0765 C15H11O2	171.0815 C12H11O	253.1234 C17H17O2
5k	8-OH-CBNA	C5H11	COOH	H	OH	12.06	369.1707 C22H25O5	295.1340 C19H19O3	187.0765 C12H11O2	325.1809 C21H25O3

[a]Analytical standard

[b]Non-isolated and identified phytocannabinoid in the literature

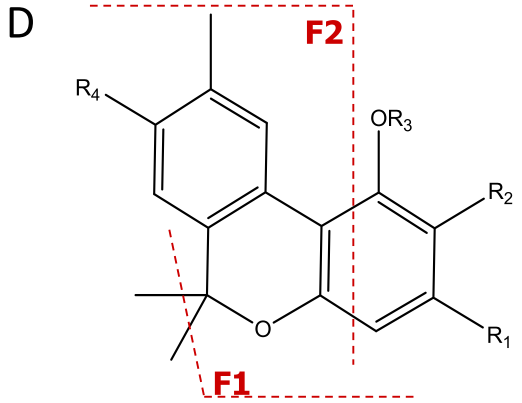

Figure S7. MS/MS spectral library of CBN-type phytocannabinoids. MS/MS spectra of the identified CBN type (**A**) neutral and (**B**) acid phytocannabinoids, (**C**) names, retention times and accurate masses, and (**D**) their fragmentation structures. Values of m/z in (**A**) and (**B**) are presented as nominal masses to improve interpretation of spectra. Accurate masses for the main fragments appear in (**C**).

A

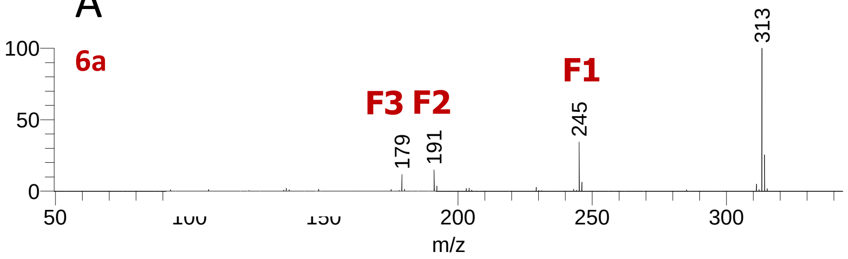

B

	Name	RT [min]	[M-H]-	[MF1-H]-	[MF2-H]-	[MF3-H]-
**6a**	d8-THC[a]	11.41	313.2173 C21H29O2	245.1547 C16H21O2	191.1078 C12H15O2	179.1067 C11H15O2

[a]Analytical standard

C

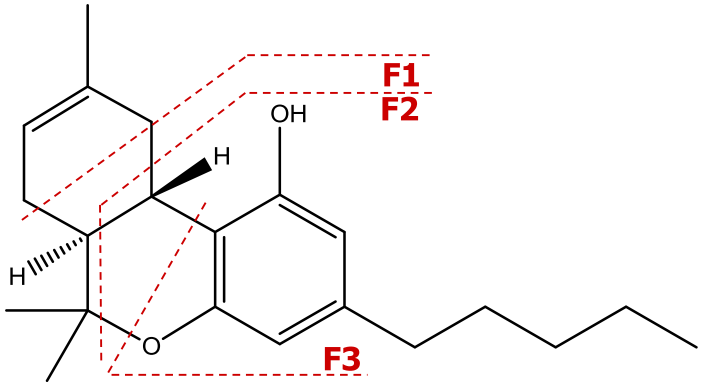

Figure S8. MS/MS spectral library of Δ⁸-THC. (**A**) MS/MS spectrum of Δ⁸-THC, (**B**) name, retention time and accurate mass, and (**C**) its fragmentation structure. Values of m/z in (**A**) are presented as nominal masses to improve interpretation of spectra. Accurate masses for the main fragments appear in (**B**).

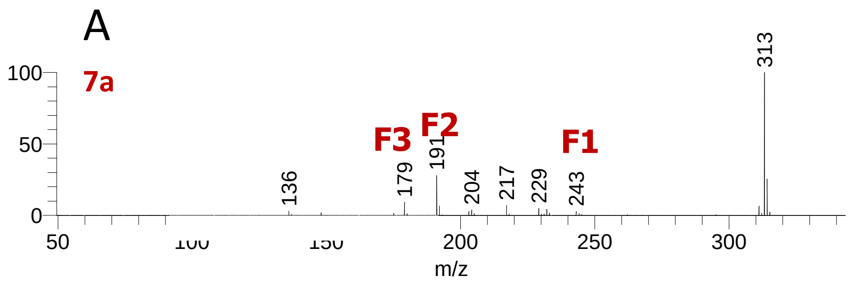

B

	Name	RT [min]	[M-H]-	[MF1-H]-	[MF2-H]-	[MF3-H]-
**7a**	CBL[a]	11.83	313.2173<br>C21H29O2	243.1391<br>C16H19O2	191.1078<br>C12H15O2	179.1067<br>C11H15O2

[a]Analytical standard

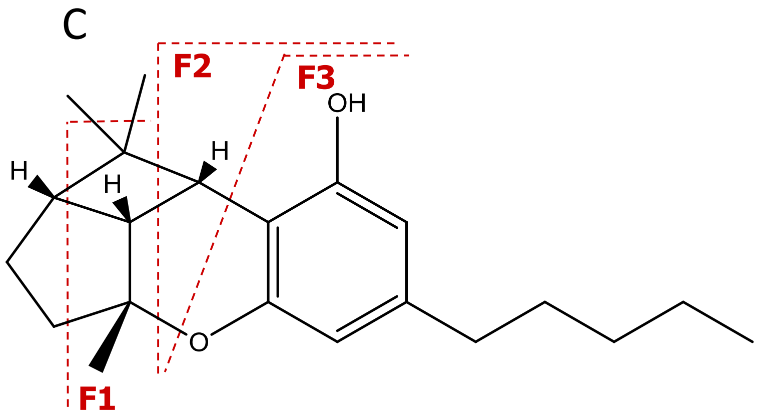

Figure S9. MS/MS spectral library of CBL. (**A**) MS/MS spectrum of CBL, (**B**) name, retention time and accurate mass, and (**C**) its fragmentation structure. Values of m/z in (**A**) are presented as nominal masses to improve interpretation of spectra. Accurate masses for the main fragments appear in (**B**).

C

	Name	R1	R2	RT [min]	[M-H]-	[MF1-H]-	[MF2-H]-	[MF3-H]-
**8a**	CBND	C5H11	H	5.79	309.1860<br>C21H25O2	279.1391<br>C19H19O2	171.0815<br>C12H11O	-
**8b**	CBNDA[a]	C5H11	COOH	5.38	353.1758<br>C22H25O4	279.1391<br>C19H19O2	171.0815<br>C12H11O	309.1860<br>C21H25O
**8c**	CBNDVA[a]	C3H7	COOH	4.22	325.1445<br>C20H21O4	251.1078<br>C17H15O2	171.0815<br>C12H11O	281.1547<br>C19H21O2

[a]Non-isolated and identified phytocannabinoid in the literature

A

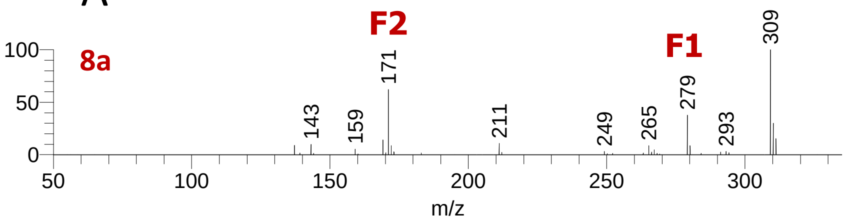

B

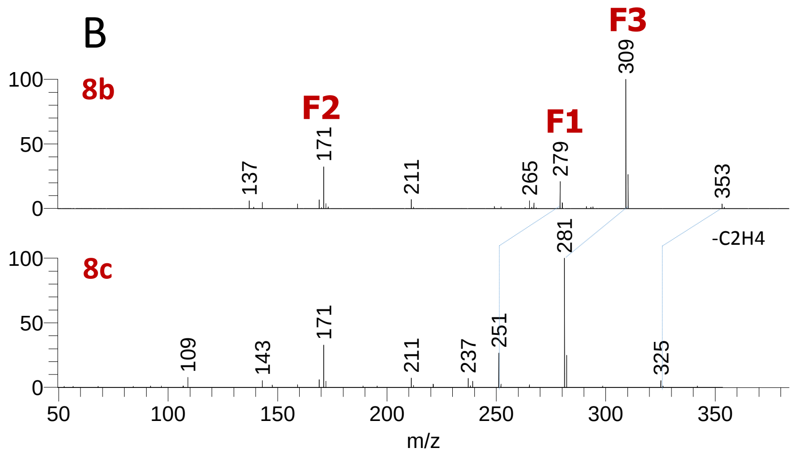

D

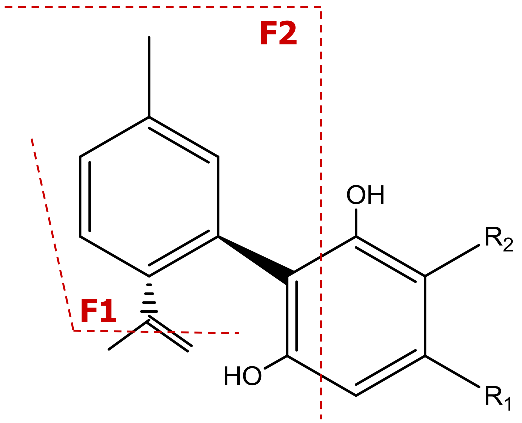

Figure S10. MS/MS spectral library of CBND-type phytocannabinoids. MS/MS spectra of the identified CBND type (**A**) neutral and (**B**) acid phytocannabinoids, (**C**) names, retention times and accurate masses, and (**D**) their fragmentation structures. Values of m/z in (**A**) and (**B**) are presented as nominal masses to improve interpretation of spectra. Accurate masses for the main fragments appear in (**C**).

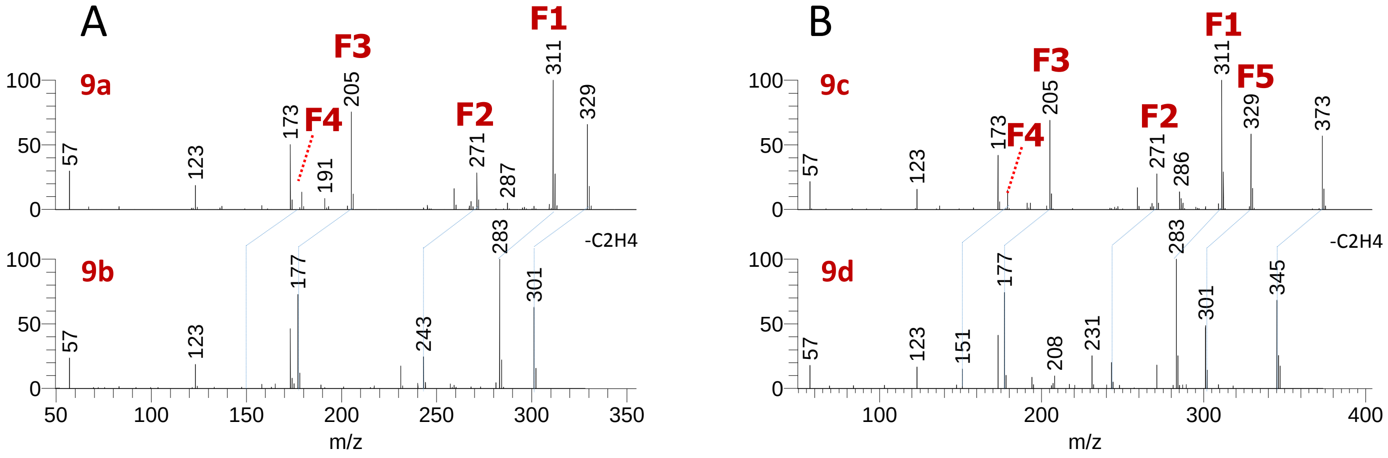

C

	Name	R1	R2	RT [min]	[M-H]-	[MF1-H]-	[MF2-H]-	[MF3-H]-	[MF4-H]-	[MF5-H]-
**9a**	CBE	C5H11	H	6.64	329.2122<br>C21H29O3	311.2017<br>C21H27O2	271.1704<br>C18H23O2	205.1234<br>C13H17O2	179.1067<br>C11H15O2	-
**9b**	CBEV	C3H7	H	4.80	301.1809<br>C19H25O3	283.1704<br>C19H23O2	243.1391<br>C16H19O2	177.0921<br>C11H13O2	151.0765<br>C9H11O2	-
**9c**	CBEA	C5H11	COOH	6.74	373.2021<br>C21H29O5	311.2017<br>C21H27O2	271.1704<br>C18H23O2	205.1234<br>C13H17O2	179.1067<br>C11H15O2	329.2122<br>C21H29O3
**9d**	CBEVA	C3H7	COOH	4.79	345.1707<br>C19H29O5	283.1704<br>C19H23O2	243.1391<br>C16H19O2	177.0921<br>C11H13O2	151.0765<br>C9H11O2	301.1809<br>C19H25O3

D

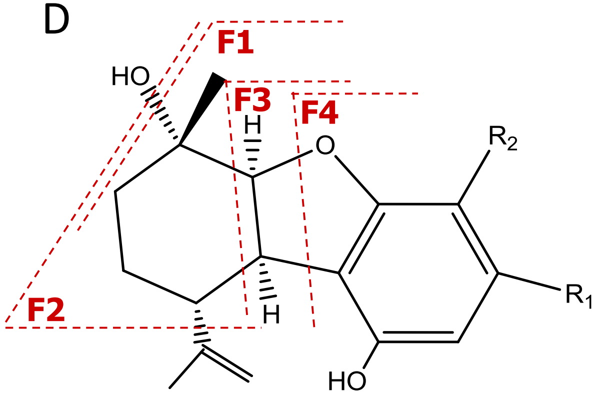

Figure S11. MS/MS spectral library of CBE-type phytocannabinoids. MS/MS spectra of the identified CBE type (**A**) neutral and (**B**) acid phytocannabinoids, (**C**) names, retention times and accurate masses, and (**D**) their fragmentation structures. Values of m/z in (**A**) and (**B**) are presented as nominal masses to improve interpretation of spectra. Accurate masses for the main fragments appear in (**C**).

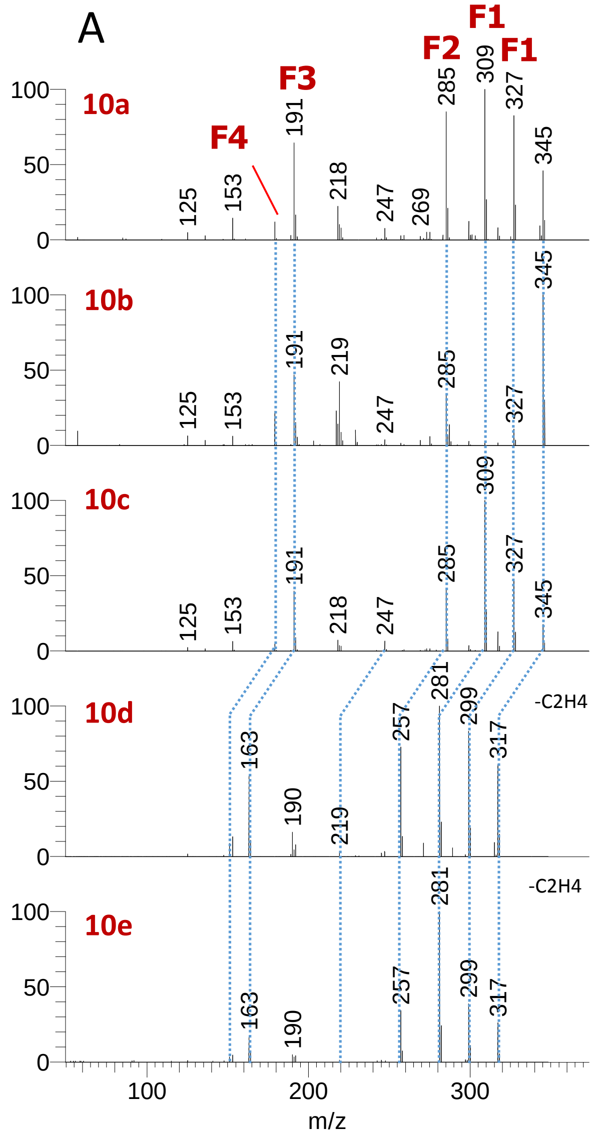

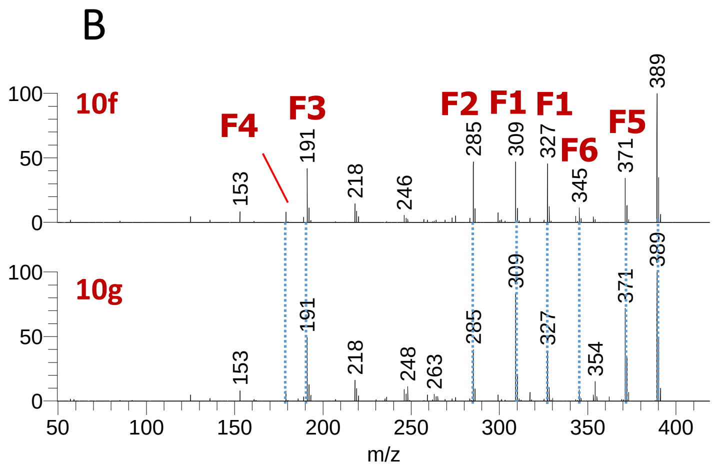

C

	Name	R1	R2	RT [min]	[M-H]-	[MF1-H]-	[MF2-H]-	[MF3-H]-	[MF4-H]-	[MF5-H]-	[MF6-H]-
**10a**	CBT isomer-1	C5H11	H	5.00	345.2071 C21H29O4	327.1966, 309.1860 C21H27O3, C21H25O2	285.1860 C19H25O2	191.1078 C12H15O2	179.1067 C11H15O2	-	-
**10b**	CBT isomer-2	C5H11	H	5.15	345.2071 C21H29O4	327.1966, 309.1860 C21H27O3, C21H25O2	285.1860 C19H25O2	191.1078 C12H15O2	179.1067 C11H15O2	-	-
**10c**	CBT isomer-3	C5H11	H	6.16	345.2071 C21H29O4	327.1966, 309.1860 C21H27O3, C21H25O2	285.1860 C19H25O2	191.1078 C12H15O2	179.1067 C11H15O2	-	-
**10d**	CBTV isomer-1	C3H7	H	3.20	317.1758 C19H25O4	299.1653, 283.1704 C19H23O3, C19H21O2	257.1547 C17H21O2	163.0765 C10H11O2	151.0765 C9H11O2	-	-
**10e**	CBTV isomer-3	C3H7	H	4.21	317.1758 C19H25O4	299.1653, 283.1704 C19H23O3, C19H21O2	257.1547 C17H21O2	163.0765 C10H11O2	151.0765 C9H11O2	-	-
**10f**	CBTA isomer-1[a]	C5H11	COOH	4.93	389.1970 C22H29O6	327.1966, 309.1860 C22H27O5, C22H25O4	285.1860 C19H25O2	191.1078 C12H15O2	179.1067 C11H15O2	371.1864 C22H27O5	345.2071 C21H29O4
**10g**	CBTA isomer-3[a]	C5H11	COOH	6.00	389.1970 C22H29O6	327.1966, 309.1860 C22H27O5, C22H25O4	285.1860 C19H25O2	191.1078 C12H15O2	179.1067 C11H15O2	371.1864 C22H27O5	345.2071 C21H29O4

[a]Non-isolated and identified phytocannabinoid in the literature

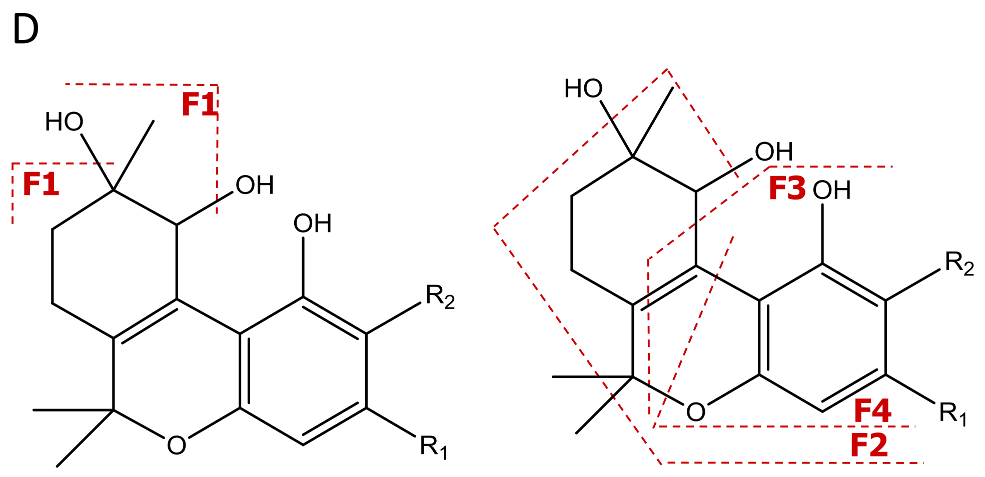

Figure S12. MS/MS spectral library of CBT-type phytocannabinoids. MS/MS spectra of the identified CBT type (**A**) neutral and (**B**) acid phytocannabinoids, (**C**) names, retention times and accurate masses, and (**D**) their fragmentation structures. Values of m/z in (**A**) and (**B**) are presented as nominal masses to improve interpretation of spectra. Accurate masses for the main fragments appear in (**C**).

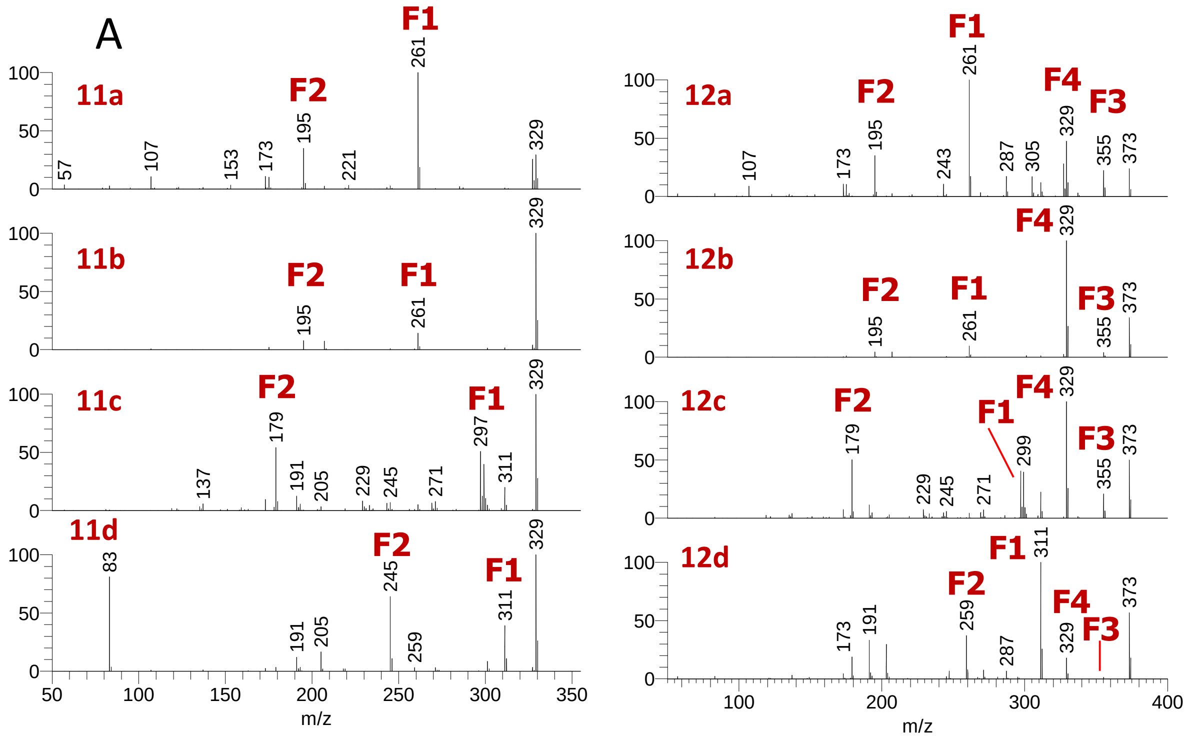

B

	Chemical formula	Acid-neutral pairing	RT [min]	[M-H]-	[MF1-H]-	[MF2-H]-	[MF3-H]-	[MF4-H]-
**11a**	C21H30O3	A	4.04	329.2122 C21H29O3	261.1496 C16H21O3	195.1027 C11H15O3	-	-
**11b**	C21H30O3	B	5.06	329.2122 C21H29O3	261.1496 C16H21O3	195.1027 C11H15O3	-	-
**11c**	C21H30O3	C	5.15	329.2122 C21H29O3	297.1860 C20H25O2	179.1078 C11H15O2	-	-
**11d**	C21H30O3	-	9.26	329.2122 C21H29O3	311.2017 C21H27O2	245.1547 C16H21O2	-	-
**12a**	C22H30O5	A	4.41	373.2021 C22H29O5	261.1496 C16H21O3	195.1027 C11H15O3	355.1915 C22H27O4	329.2122 C21H29O3
**12b**	C22H30O5	B	6.33	373.2021 C22H29O5	261.1496 C16H21O3	195.1027 C11H15O3	355.1915 C22H27O4	329.2122 C21H29O3
**12c**	C22H30O5	C	5.64	373.2021 C22H29O5	297.1860 C20H25O2	179.1078 C11H15O2	355.1915 C22H27O4	329.2122 C21H29O3
**12d**	C22H30O5	-	9.83	373.2021 C22H29O5	311.2017 C21H27O2	259.1704 C16H21O2	355.1915 C22H27O4	329.2122 C21H29O3

Figure S13. MS/MS spectral library of additional phytocannabinoids (Part I). (**A**) MS/MS spectra, and (**B**) names, retention times and accurate masses. Values of m/z in (**A**) are presented as nominal masses to improve interpretation of spectra. Accurate masses for the main fragments appear in (**B**).

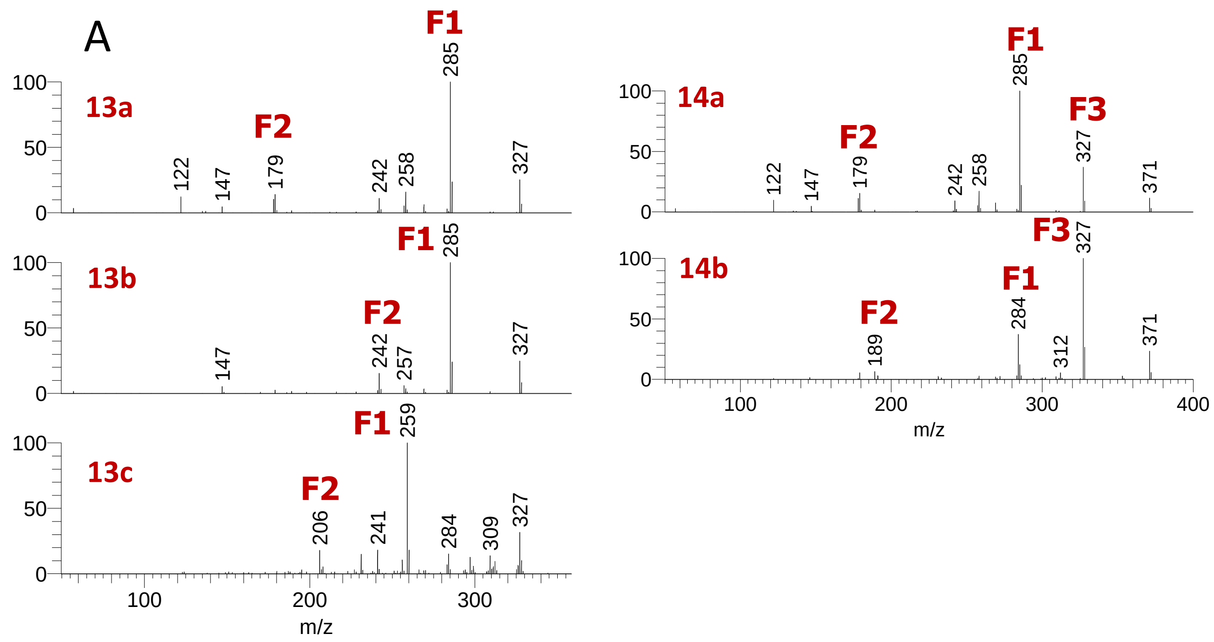

B

	Chemical formula	Acid-neutral pairing	RT [min]	[M-H]-	[MF1-H]-	[MF2-H]-	[MF3-H]-
**13a**	$C_{21}H_{28}O_3$	A	6.49	327.1966<br>$C_{21}H_{27}O_3$	285.1860<br>$C_{19}H_{25}O_2$	179.1078<br>$C_{11}H_{15}O_2$	-
**13b**	$C_{21}H_{28}O_3$	-	5.97	327.1966<br>$C_{21}H_{27}O_3$	285.1860<br>$C_{19}H_{25}O_2$	242.1312<br>$C_{16}H_{18}O_2$	-
**13c**	$C_{21}H_{28}O_3$	-	11.06	327.1966<br>$C_{21}H_{27}O_3$	259.1340<br>$C_{16}H_{19}O_3$	206.0948<br>$C_{12}H_{14}O_3$	-
**14a**	$C_{22}H_{28}O_5$	A	6.75	371.1864<br>$C_{22}H_{27}O_5$	285.1860<br>$C_{19}H_{25}O_2$	179.1078<br>$C_{11}H_{15}O_2$	327.1966<br>$C_{21}H_{27}O_3$
**14b**	$C_{22}H_{28}O_5$	-	8.04	371.1864<br>$C_{22}H_{27}O_5$	284.1418<br>$C_{18}H_{20}O_3$	189.0921<br>$C_{12}H_{13}O_2$	327.1966<br>$C_{21}H_{27}O_3$

Figure S13. MS/MS spectral library of additional phytocannabinoids (Part II). (**A**) MS/MS spectra, and (**B**) names, retention times and accurate masses. Values of m/z in (**A**) are presented as nominal masses to improve interpretation of spectra. Accurate masses for the main fragments appear in (**B**).

A

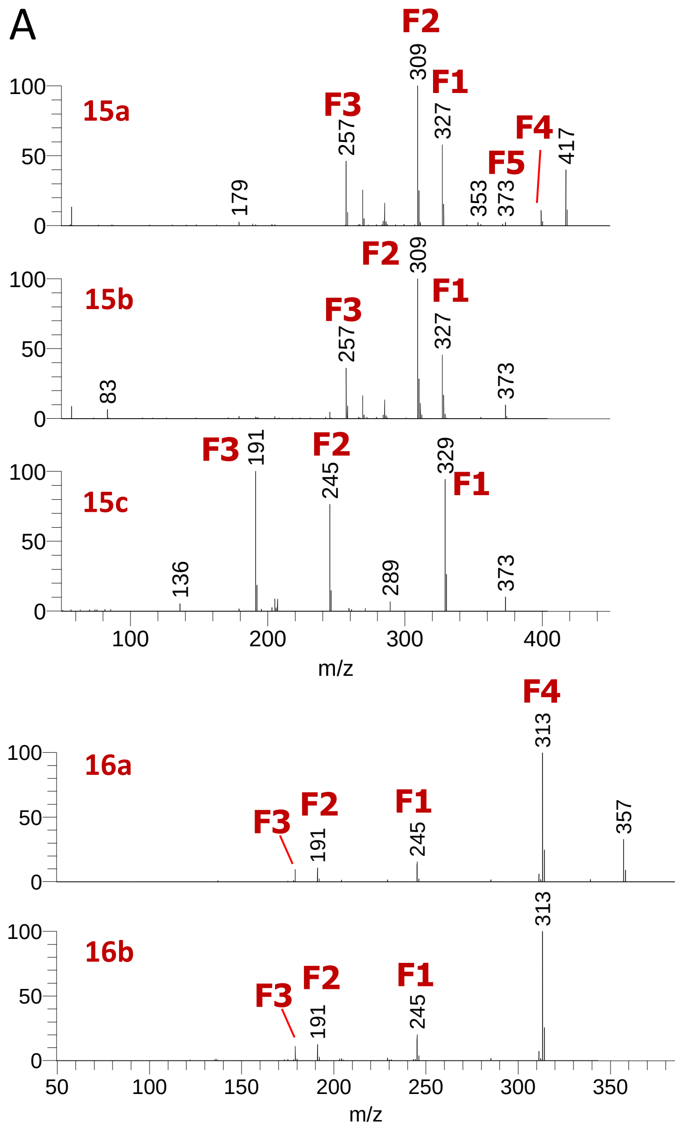

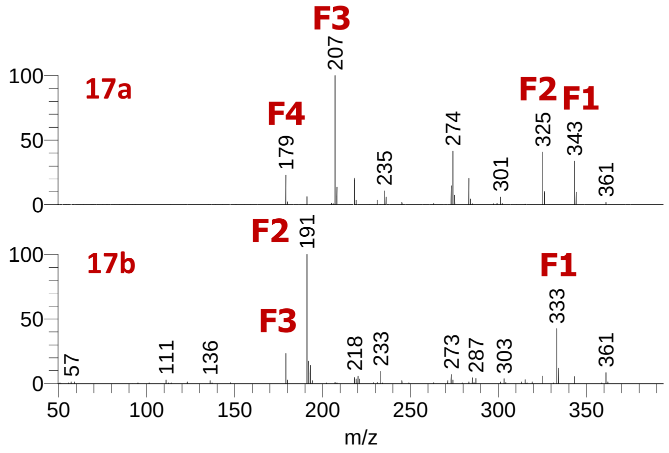

B

	Chemical formula	Acid-neutral pairing	RT [min]	[M-H]-	[MF1-H]-	[MF2-H]-	[MF3-H]-	[MF4-H]-	[MF5-H]-
**15a**	C24H34O6	A	6.2	417.2283<br>C24H33O6	327.1966<br>C21H27O3	309.1860<br>C21H25O2	257.1547<br>C17H21O2	399.2177<br>C24H31O5	373.2384<br>C23H33O4
**15b**	C23H34O4	A	7.48	373.2384<br>C23H33O4	327.1966<br>C21H27O3	309.1860<br>C21H25O2	257.1547<br>C17H21O2	-	-
**15c**	C23H34O4	-	13.15	373.2384<br>C23H33O4	329.2486<br>C22H33O2	245.1547<br>C16H21O2	191.1078<br>C12H15O2	-	-
**16a**	C22H30O4	B	12.31	357.2071<br>C22H29O4	245.1547<br>C16H21O2	191.1078<br>C12H15O2	179.1067<br>C11H15O2	-	313.2173<br>C21H29O2
**16b**	C21H30O2	B	10.83	313.2173<br>C21H29O2	245.1547<br>C16H21O2	191.1078<br>C12H15O2	179.1067<br>C11H15O2	-	-
**17a**	C21H30O5	-	6.43	361.2021<br>C21H29O5	343.1915<br>C21H27O4	325.1809<br>C21H25O3	207.1027<br>C12H15O3	179.1067<br>C11H15O2	-
**17b**	C21H30O5	-	3.64	361.2021<br>C21H29O5	333.2071<br>C20H29O4	191.1078<br>C12H15O2	179.1067<br>C11H15O2	-	-

Figure S13. MS/MS spectral library of additional phytocannabinoids (Part III). (**A**) MS/MS spectra, and (**B**) names, retention times and accurate masses. Values of m/z in (**A**) are presented as nominal masses to improve interpretation of spectra. Accurate masses for the main fragments appear in (**B**).

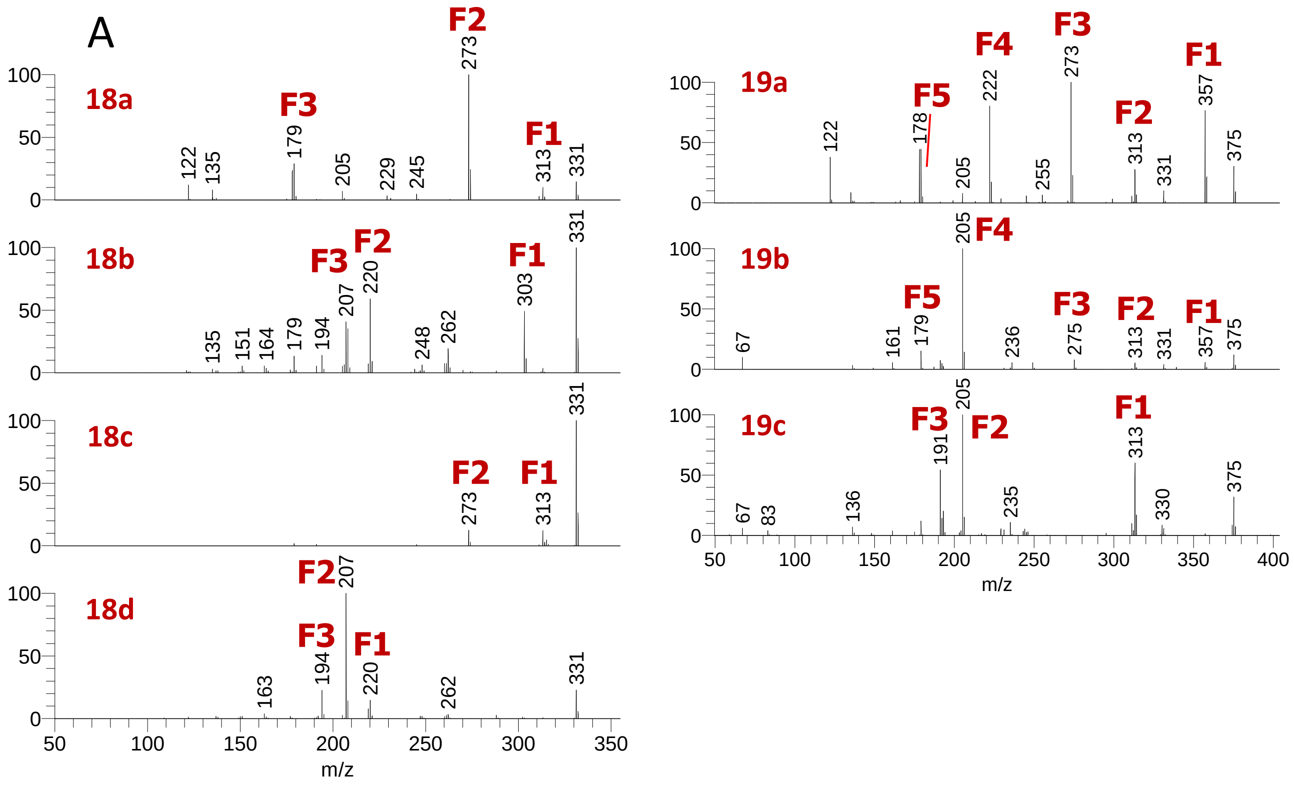

B

	Chemical formula	RT [min]	[M-H]-	[MF1-H]-	[MF2-H]-	[MF3-H]-	[MF4-H]-	[MF5-H]-
**18a**	$C_{21}H_{32}O_3$	5.60	331.2279<br>$C_{21}H_{31}O_3$	313.2173<br>$C_{21}H_{29}O_2$	273.1860<br>$C_{18}H_{25}O_2$	179.1067<br>$C_{11}H_{15}O_2$	-	-
**18b**	$C_{21}H_{32}O_3$	7.58	331.2279<br>$C_{21}H_{31}O_3$	303.2330<br>$C_{20}H_{31}O_2$	220.1105<br>$C_{13}H_{16}O_3$	207.1027<br>$C_{12}H_{15}O_3$	-	-
**18c**	$C_{21}H_{32}O_3$	8.74	331.2279<br>$C_{21}H_{31}O_3$	313.2173<br>$C_{21}H_{29}O_2$	273.1860<br>$C_{18}H_{25}O_2$	-	-	-
**18d**	$C_{21}H_{32}O_3$	10.12	331.2279<br>$C_{21}H_{31}O_3$	220.1105<br>$C_{13}H_{16}O_3$	207.1027<br>$C_{12}H_{15}O_3$	194.0948<br>$C_{11}H_{14}O_3$	-	-
**19a**	$C_{22}H_{32}O_5$	4.80	375.2177<br>$C_{22}H_{31}O_5$	357.2071<br>$C_{22}H_{29}O_4$	313.2173<br>$C_{21}H_{29}O_2$	273.1860<br>$C_{18}H_{25}O_2$	222.0898<br>$C_{12}H_{14}O_4$	179.1067<br>$C_{11}H_{15}O_2$
**19b**	$C_{22}H_{32}O_5$	3.95	375.2177<br>$C_{22}H_{31}O_5$	357.2071<br>$C_{22}H_{29}O_4$	313.2173<br>$C_{21}H_{29}O_2$	275.2017<br>$C_{13}H_{17}O_2$	205.1234<br>$C_{13}H_{17}O_2$	179.1067<br>$C_{11}H_{15}O_2$
**19c**	$C_{22}H_{32}O_5$	9.06	375.2177<br>$C_{22}H_{31}O_5$	313.2173<br>$C_{21}H_{29}O_2$	205.1234<br>$C_{13}H_{17}O_2$	191.1078<br>$C_{12}H_{15}O_2$	-	-

Figure S13. MS/MS spectral library of additional phytocannabinoids (Part IV). (**A**) MS/MS spectra, and (**B**) names, retention times and accurate masses. Values of m/z in (**A**) are presented as nominal masses to improve interpretation of spectra. Accurate masses for the main fragments appear in (**B**).

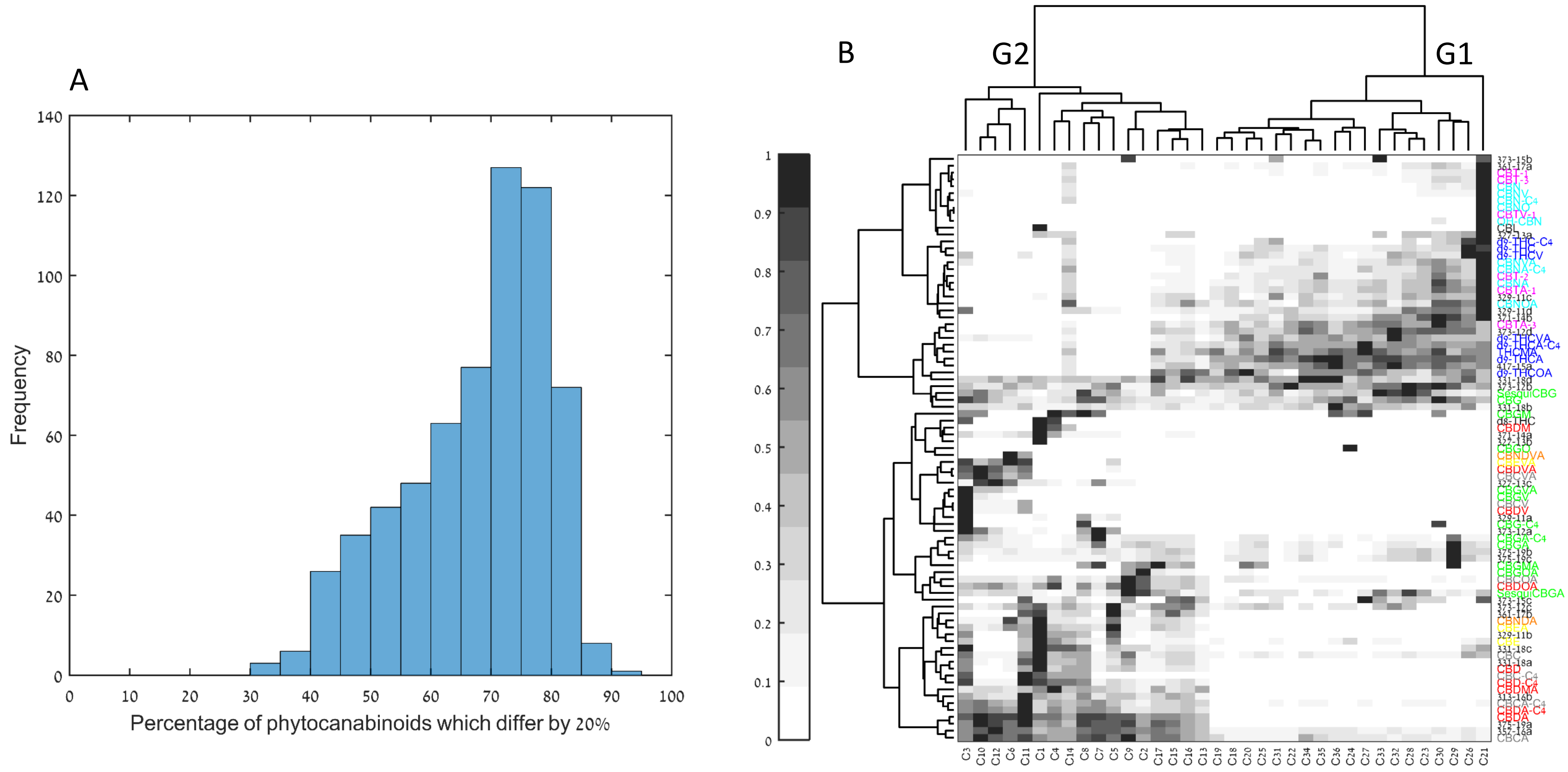

Figure S14. Data analysis methods for exploring variations of phytocannabinoids for the 36 *Cannabis* samples. (**A**) Histogram of the percent of phytocannabinoid profiles for every pair of *Cannabis* samples that differ by more than 20% in concentration. (**B**) Heat map of the LC-MS normalized data of the 36 *Cannabis* samples organized according to hierarchical clustering of both *Cannabis* samples and phytocannabinoids. Each phytocannabinoid subclass is marked by a different color. The largest variation between *Cannabis* samples can be attributed to the content of Δ^9-THC- (G1) and CBD-type (G2) phytocannabinoids and decomposition products.

Table S1. List of neutral and acid potential masses of phytocannabinoids used in data dependent MS/MS mode

	**Neutral-type phytocannabinoids**			**Acid-type phytocannabinoids**		
Phytocannabinoid subclass	Name	Chemical Formula	$[M-H]^-$	Name	Chemical Formula	$[M-H]^-$
CBN	Cannabiorcol-C1 (CBNO)	C17H18O2	253.12340	Cannabiorcolic acid-c1 (CBNOA)*	C18H18O4	297.11323
CBND	CBND-C1 (CBNDO)*	C17H18O2	253.12340	CBNDA-C1 (CBNDOA)*	C18H18O4	297.11323
Δ^9-THC	(-)-Δ^9- *trans*-Tetrahydrocannabiorcol-C1 (Δ^9-THCO)	C17H22O2	257.15470	(-)-Δ^9- *trans*-Tetrahydrocannabiorcolic acid-C1 (Δ^9-THCOA)	C18H22O4	301.14453
CBD	Cannabidiorcol-C1 (CBDO)	C17H22O2	257.15470	Cannabidiorcolic acid-C1 (CBDOA)*	C18H22O4	301.14453
CBC	Cannabiorchromene-C1 (CBCO)	C17H22O2	257.15470	Cannabiorchromenic acid-C1 (CBCOA)	C18H22O4	301.14453
Δ^8-THC	(-)-Δ^8-*trans*-(6aR,10aR)-Tetrahydrocannabiorcol-C1 (Δ^8-THCO)*	C17H22O2	257.15470	(-)-Δ^8-*trans*-(6aR,10aR)-Tetrahydrocannabiorcolic acid-C1 (Δ^8-THCOA)*	C18H22O4	301.14453
CBL	Cannabiorcyclol C1 (CBLO)	C17H22O2	257.15470	Cannabiorcyclolic acid C1 (CBLOA)	C18H22O4	301.14453
CBG	CBG-C1 (CBGO)*	C17H24O2	259.17035	CBGA-C1 (CBGOA)*	C18H24O4	303.16018
CBN	Cannabinol-C2 (CBN-C2)	C18H20O2	267.13905	CBNA-C2*	C19H20O4	311.12888
CBND	CBND-C2*	C18H20O2	267.13905	CBNDA-C2*	C19H20O4	311.12888
Δ^9-THC	Δ^9-THC-C2*	C18H24O2	271.17035	Δ^9-THCA-C2*	C19H24O4	315.16018
CBD	CBD-C2*	C18H24O2	271.17035	CBDA-C2*	C19H24O4	315.16018
CBC	CBC-C2*	C18H24O2	271.17035	CBCA-C2*	C19H24O4	315.16018
Δ^8-THC	Δ^8-THC-C2*	C18H24O2	271.17035	Δ^8-THCA-C2*	C19H24O4	315.16018
CBL	CBL-C2*	C18H24O2	271.17035	CBLA-C2*	C19H24O4	315.16018
CBE	Bisnor-cannabielsoin-C1 (CBEO)	C17H22O3	273.14962	Bisnor-cannabielsoinic acid-C1 (CBEOA)	C18H22O5	317.13945
CBG	CBG-C2*	C18H26O2	273.18600	CBGA-C2*	C19H26O4	317.17583
CBN	Cannabivarin-C3 (CBNV)	C19H22O2	281.15470	CBNA-C3 (CBNVA)*	C20H22O4	325.14453
CBND	Cannabinodivarin-C3 (CBNDV)	C19H22O2	281.15470	CBNDA-C3 (CBNDVA)*	C20H22O4	325.14453
Δ^9-THC	(-)-Δ^9-*trans*-Tetrahydrocannabivarin-C3 (Δ^9-THCV)	C19H26O2	285.18600	(-)-Δ^9-*trans*-Tetrahydrocannabivarinic acid-C3 (Δ^9-THCVA)	C20H26O4	329.17583

CBD	(-)-Cannabidivarin-C3 (CBDV)	C19H26O2	285.18600	Cannabidivarinic acid-C3 (CBDVA)	C20H26O4	329.17583
CBC	(±)-Cannabichromevarin-C3 (CBCV)	C19H26O2	285.18600	(±)-Cannabichromevarinic acid-C3 (CBCVA)	C20H26O4	329.17583
Δ^8-THC	(-)-Δ^8-*trans*-THC-C3 (Δ^8-THCV)*	C19H26O2	285.18600	(-)-Δ^8-*trans*-THCA-C3 (Δ^8-THCVA)*	C20H26O4	329.17583
CBL	(±)-(1aS,3aR,8bR,8cR)-Cannabicyclovarin-C3 (CBLV)	C19H26O2	285.18600	Cannabicyclolyc acid-C3 (CBLVA)	C20H26O4	329.17583
CBC	2-Methyl-2-(4-methyl-2-pentenyl)-7-propyl-2H-1-benzopyran-5-ol	C19H26O2	285.18600	2-Methyl-2-(4-methyl-2-pentenyl)-7-propyl-2H-1-benzopyran-5-ol acid*	C20H26O4	329.17583
Miscellaneous	Δ^7-tetrahydrocannabivarin-C3 (Δ^7-THCV)	C19H26O2	285.18600	Δ^7-tetrahydrocannabivarinic acid-C3 (Δ^7-THCVA)*	C20H26O4	329.17583
CBE	CBE-C2*	C18H24O3	287.16527	CBEA-C2*	C19H24O5	331.15510
CBG	Cannabigerovarin-C3 (CBGV)	C19H28O2	287.20165	Cannabigerovarinic acid-C3 (CBGVA)	C20H28O4	331.19148
CBT	Cannabitriol-C1 (CBTO)	C17H22O4	289.14453	Cannabitriolic acid-C1 (CBTOA)*	C18H22O6	333.13436
CBN	Cannabinol-C4 (CBN-C4)	C20H24O2	295.17035	Cannabinolic acid-C4 (CBNA-C4)*	C21H24O4	339.16018
CBND	CBND-C4*	C20H24O4	295.17035	CBNDA-C4*	C21H24O4	339.16018
Δ^9-THC	(-)-Δ^9-*trans*-Tetrahydrocannabinol-C4 (Δ^9-THC-C4)	C20H28O2	299.20165	(-)-Δ^9-*trans*-Tetrahydrocannabinolic acid-C4 (Δ^9-THCA-C4)	C21H28O4	343.19148
CBD	Cannabidiol-C4 (CBD-C4)	C20H28O2	299.20165	CBDA-C4*	C21H28O4	343.19148
CBC	CBC-C4*	C20H28O2	299.20165	CBCA-C4*	C21H28O4	343.19148
Δ^8-THC	(-)-*trans*-Δ^8-THC-C4*	C20H28O2	299.20165	(-)-*trans*-Δ^8-THCA-C4*	C21H28O4	343.19148
CBL	CBL-C4*	C20H28O2	299.20165	CBLA-C4*	C21H28O4	343.19148
CBE	Cannabielsoin-C3 (CBEV)	C19H26O3	301.18092	Cannabielsoic acid-C3 (CBEVA)	C20H26O5	345.17075
CBG	CBG-C4*	C20H30O2	301.21730	CBGA-C4*	C21H30O4	345.20713
CBT	CBT-C2*	C18H24O4	303.16018	CBTA-C2*	C19H24O6	347.15001
Miscellaneous	Cannabichromanone-C3	C18H24O4	303.16018	Cannabichromanonic acid-C3*	C19H24O6	347.15001
Miscellaneous	Cannabiglendol-C3 (OH-iso-HHCV-C3)	C19H28O3	303.19657	Cannabiglendolic acid-C3 (OH-iso-HHCVA-C3)*	C20H28O5	347.18640
Miscellaneous	Cannabioxepane-C5 (CBX)	C21H22O2	305.15470	Cannabioxepanic acid-C5 (CBXA)*	C22H22O4	349.14453
Miscellaneous	Dehydrocannabifuran-C5 (DCBF)	C21H24O2	307.17035	Dehydrocannabifuranic acid-C5 (DCBFA)*	C22H24O4	351.16018
CBN	Cannabinol-C5 (CBN)	C21H26O2	309.18600	Cannabinolic acid-C5 (CBNA)	C22H26O4	353.17583
CBND	Cannabinodiol-C5 (CBND)	C21H26O2	309.18600	Cannabinodiolic acid-C5 (CBNDA)*	C22H26O4	353.17583

Miscellaneous	Cannabifuran-C5 (CBF)	C21H26O2	309.18600	Cannabifuranic acid-C5 (CBFA)*	C22H26O4	353.17583
Δ^9-THC	(-)-Δ^9-*trans*-Tetrahydrocannabinol-C5 (Δ^9-THC)	C21H30O2	313.21730	(-)-Δ^9-*trans*-Tetrahydrocannabinolic acid-C5 (Δ^9-THCA)	C22H30O4	357.20713
Δ^8-THC	(-)-Δ^8-*trans*-(6aR,10aR)-Tetrahydrocannabinol-C5 (Δ^8-THC)	C21H30O2	313.21730	(-)-Δ^8-*trans*-(6aR,10aR)-Tetrahydrocannabinolic acid-C5 (Δ^8-THCA)	C22H30O4	357.20713
CBC	(±)-Cannabichromene-C5 (CBC)	C21H30O2	313.21730	(±)-Cannabichromenic acid-C5 (CBCA)	C22H30O4	357.20713
CBD	(-)-Cannabidiol-C5 (CBD)	C21H30O2	313.21730	Cannabidiolic acid-C5 (CBDA)	C22H30O4	357.20713
CBL	(±)-(1aS,3aR,8bR,8cR)-Cannabicyclol-C5 (CBL)	C21H30O2	313.21730	(±)-(1aS,3aR,8bR,8cR)-Cannabicyclolic acid-C5 (CBLA)	C22H30O4	357.20713
Miscellaneous	Cannabicitran-C5 (CBR)	C21H30O2	313.21730	Cannabicitranic acid-C5 (CBRA)*	C22H30O4	357.20713
Miscellaneous	(-)-Δ^9-(6aS,10aR-*cis*)-Tetrahydrocannabinol-C5 ((-)-*cis*-Δ^9-THC)	C21H30O2	313.21730	(-)-Δ^9-(6aS,10aR-*cis*)-Tetrahydrocannabinolic acid-C5 ((-)-*cis*-Δ^9-THCA)*	C22H30O5	357.20713
Miscellaneous	(-)-Δ^7-*trans*-(1R,3R,6R)-Isotetrahydrocannabinol-C5 (*trans*-iso-Δ^7-THC)	C21H30O2	313.21730	(-)-Δ^7-*trans*-(1R,3R,6R)-Isotetrahydrocannabinolic acid-C5 (*trans*-iso-Δ^7-THCA)*	C22H30O4	357.20713
CBE	CBE-C4*	C20H28O3	315.19657	CBEA-C4*	C21H28O5	359.18640
CBG	Cannabigerol-C5 (CBG)	C21H32O2	315.23295	Cannabigerolic acid-C5 (CBGA)	C22H32O4	359.22278
CBT	Cannabitriol-C3 (CBTV)	C19H26O4	317.17583	CBTA-C3 (CBTVA)*	C20H26O6	361.16566
CBN	Cannabinol methyl ether-C5 (CBNM)	C22H28O2	323.20165	CBNMA-C5 (CBNMA)*	C23H28O4	367.19148
CBND	CBNDM-C5*	C22H28O2	323.20165	CBNDMA-C5 (CBNDMA)*	C23H28O4	367.19148
CBN	8-OH-CBN-C5 (OH-CBN)	C21H26O3	325.18092	8-OH-CBNA-C5 (OH-CBNA)	C22H26O5	369.17075
CBND	OH-CBND-C5 (OH-CBND)*	C21H26O3	325.18092	OH-CBNDA-C5 (OH-CBNDA)*	C22H26O5	369.17075
Miscellaneous	10-Oxo-$\Delta^{6a(10a)}$-Tetrahydrocannabinol-C5 (OTHC)	C21H28O3	327.19657	10-Oxo-$\Delta^{6a(10a)}$-Tetrahydrocannabinolic acid-C5 (OTHCA)*	C22H28O5	371.18640
Miscellaneous	Cannabichromanone D-C5	C21H28O3	327.19657	Cannabichromanonic acid D-C5*	C22H28O5	371.18640
Miscellaneous	Cannabicoumaronone-C5 (CBCON-C5)	C21H28O3	327.19657	Cannabicoumaronic acid-C5 (CBCONA)	C22H28O5	371.18640
CBD	Cannabidiol monomethyl ether-C5 (CBDM)	C22H32O2	327.23295	CBDMA-C5 (CBDMA)*	C23H32O4	371.22278
Δ^9-THC	Δ^9-THCM-C5*	C22H32O2	327.23295	Δ^9-THCMA-C5 (Δ^9-THCMA)*	C23H32O4	371.22278
CBC	(±)-3"-hydroxy-$\Delta^{4"}$-cannabichromene-C5	C21H30O3	329.21222	(±)-3"-hydroxy-$\Delta^{4"}$-cannabichromenic acid-C5*	C22H30O5	373.20205

CBE	(5aS,6S,9R,9aR)-Cannabielsoin-C5 (CBE)	C21H30O3	329.21222	(5aS,6S,9R,9aR)-Cannabielsoic acid-C5 (CBEA)	C22H30O5	373.20205
Miscellaneous	2-geranyl-5-hydroxy-3-n-pentyl-1,4-benzoquinone-C5	C21H30O3	329.21222	2-geranyl-5-hydroxy-3-n-pentyl-1,4-benzoquinonic acid-C5*	C22H30O5	373.20205
Δ^9-THC	8α-Hydroxy-Δ^9-Tetrahydrocannabinol-C5 (8α-OH-Δ^9-THC)	C21H30O3	329.21222	8α-Hydroxy-Δ^9-Tetrahydrocannabinolic acid-C5 (8α-OH-Δ^9-THCA)*	C22H30O5	373.20205
Δ^9-THC	8β-Hydroxy-Δ^9-Tetrahydrocannabinol-C5 (8β-OH-Δ^9-THC)	C21H30O3	329.21222	8β-Hydroxy-Δ^9-Tetrahydrocannabinolic acid-C5 (8β-OH-Δ^9-THCA)*	C22H30O5	373.20205
Δ^8-THC	10α-Hydroxy-Δ^8-Tetrahydrocannabinol-C5 (10α-OH-Δ^8-THC)	C21H30O3	329.21222	10α-Hydroxy-Δ^8-Tetrahydrocannabinolic acid-C5 (10α-OH-Δ^8-THCA)*	C22H30O5	373.20205
Δ^8-THC	10β-Hydroxy-Δ^8-Tetrahydrocannabinol-C5 (10β-OH-Δ^8-THC)	C21H30O3	329.21222	10β-Hydroxy-Δ^8-Tetrahydrocannabinolic acid-C5 (10β-OH-Δ^8-THCA)*	C22H30O5	373.20205
Δ^9-THC	10α-hydroxy-$\Delta^{9,11}$-hexahydrocannabinol-C5	C21H30O3	329.21222	10α-hydroxy-$\Delta^{9,11}$-hexahydrocannabinolic acid-C5*	C22H30O5	373.20205
Δ^9-THC	9β,10β-Epoxyhexahydrocannabinol-C5	C21H30O3	329.21222	9β,10β-Epoxyhexahydrocannabinolic acid-C5*	C22H30O5	373.20205
CBD	OH-CBD-C5 (OH-CBD)*	C21H30O3	329.21222	OH-CBDA-C5 (OH-CBDA)*	C22H30O5	373.20205
CBG	Cannabigerol monomethyl ether-C5 (CBGM)	C22H34O2	329.24860	Cannabigerolic acid monomethyl ether-C5 (CBGMA)	C23H34O4	373.23843
Miscellaneous	Cannabichromanone-C5	C20H28O4	331.19148	Cannabichromanonic acid-C5*	C21H28O6	375.18131
CBT	CBT-C4*	C20H28O4	331.19148	CBTA-C4*	C21H28O6	375.18131
CBG	(±)-6,7-*cis*-epoxycannabigerol-C5	C21H32O3	331.22787	(±)-6,7-*trans*-epoxycannabigerolic acid-C5	C22H32O5	375.21770
CBG	(±)-6,7-*trans*-epoxycannabigerol-C5	C21H32O3	331.22787	(±)-6,7-*cis*-epoxycannabigerolic acid-C5	C22H32O5	375.21770
CBC	(–)-7-hydroxycannabichromane-C5	C21H32O3	331.22787	(–)-7-hydroxycannabichromanic acid-C5*	C22H32O5	375.21770
CBD	Cannabimovone-C5	C21H30O4	345.20713	Cannabimovonic acid-C5*	C22H30O6	389.19696
CBT	(-)-*trans*-Cannabitriol-C5 ((-)-*trans*-CBT)	C21H30O4	345.20713	(-)-*trans*-Cannabitriolic acid-C5 ((-)-*trans*-CBTA)*	C22H30O6	389.19696
CBT	(+)-*trans*-Cannabitriol-C5 ((+)-*trans*-CBT)	C21H30O4	345.20713	(+)-*trans*-Cannabitriolic acid-C5 ((+)-*trans*-CBTA)*	C22H30O6	389.19696
CBT	(±)-*cis*-Cannabitriol-C5 ((±)-*cis*-CBT)	C21H30O4	345.20713	(±)-*cis*-Cannabitriolic acid-C5 ((±)-*cis*-CBTA)*	C22H30O6	389.19696
CBT	(-)-*trans*-10-Ethoxy-9-hydroxy-$\Delta^{6a(10a)}$-tetrahydrocannabivarin-C3 [(-)-*trans*-CBT-OEt]	C21H30O4	345.20713	(-)-*trans*-10-Ethoxy-9-hydroxy-$\Delta^{6a(10a)}$-tetrahydrocannabivarinic acid-C3 ((-)-*trans*-CBT-OEt)*	C22H30O6	389.19696

Miscellaneous	(-)-(6aR,9S,10S,10aR)-9,10-Dihydroxyhexahydrocannabinol-C5 [(-)-Cannabiripsol] (CBR)	C21H32O4	347.22278	(-)-(6aR,9S,10S,10aR)-9,10-Dihydroxyhexahydrocannabinolic acid-C5 [(-)-Cannabiripsolic acid] (CBRA)*	C22H32O6	391.21261
Miscellaneous	Cannabichromanone C-C5	C21H28O5	359.18640	Cannabichromanonic acid C-C5*	C22H28O7	403.17623
Miscellaneous	(-)-6a,7,10a-Trihydroxy-Δ^9-tetrahydrocannabinol-C5 [(-)-Cannabitetrol] (CBTT)	C21H30O5	361.20205	(-)-6a,7,10a-Trihydroxy-Δ^9-tetrahydrocannabinolic acid-C5 ((-)-Cannabitetrol) (CBTTA)*	C22H30O7	405.19188
Miscellaneous	Cannabichromanone B-C5	C21H30O5	361.20205	Cannabichromanonic acid B-C5*	C22H30O7	405.19188
CBT	8,9-Dihydroxy-$\Delta^{6a(10a)}$-tetrahydrocannabinol-C5 (8,9-Di-OH-CBT)	C21H30O5	361.20205	8,9-Dihydroxy-$\Delta^{6a(10a)}$-tetrahydrocannabinolic acid-C5 (8,9-Di-OH-CBT)*	C22H30O7	405.19188
CBC	(±)-4-acetoxycannabichromene-C5	C23H32O4	371.22278	(±)-4-acetoxycannabichromenic acid-C5*	C24H32O6	415.21261
Miscellaneous	2-acetoxy-6-geranyl-3-n-pentyl-1,4-benzoquinone-C5	C23H32O4	371.22278	2-acetoxy-6-geranyl-3-n-pentyl-1,4-benzoquinonic acid-C5*	C24H32O6	415.21261
Miscellaneous	11-Acetoxy-Δ^9-Tetrahydrocannabinol-C5 (11-OAc-Δ^9-THC)*	C23H32O5	371.22278	11-Acetoxy-Δ^9-Tetrahydrocannabinoic acid-C5 (11-OAc-Δ^9-THCA)	C24H32O6	415.21261
CBG	5-acetyl-4-hydroxycannabigerol-C5	C23H34O4	373.23843	5-acetyl-4-hydroxycannabigerolic acid-C5*	C24H34O6	417.22826
CBG	4-acetoxy-2-geranyl-5-hydroxy-3-n-pentylphenol-C5	C23H34O4	373.23843	4-acetoxy-2-geranyl-5-hydroxy-3-n-pentylphenolic acid-C5*	C24H34O6	417.22826
CBT	(-)-*trans*-10-Ethoxy-9-hydroxy-$\Delta^{6a(10a)}$-tetrahydrocannabinol-C5 ((-)-*trans*-CBT-Oet)	C23H34O4	373.23843	(-)-*trans*-10-Ethoxy-9-hydroxy-$\Delta^{6a(10a)}$-tetrahydrocannabinolic acid-C5 ((-)-*trans*-CBTA-Oet)*	C24H34O6	417.22826
	4-acetoxy-2-geranyl-5-hydroxy-3-n-propylphenol-C5	C23H34O4	373.23843	4-acetoxy-2-geranyl-5-hydroxy-3-n-propylphenolic acid-C5*	C24H34O6	417.22826
CBG	sesquicannabigerol-C5 (SesquiCBG)	C26H40O2	383.29555	Sesquicannabigerolic acid-C5 (SesquiCBGA)*	C27H40O4	427.28538
CBD	carmagerol-C5	C23H36O6	407.24391	Carmagerolic acid-C5*	C24H36O8	451.23374
CBN	4-terpenyl cannabinolate-C5	C32H42O4	489.30103			
Δ^9-THC	β-fenchyl-Δ^9-tetrahydrocannabinolate-C5	C32H46O4	493.33233			
Δ^9-THC	α-fenchyl-Δ^9-tetrahydrocannabinolate-C5	C32H46O4	493.33233			
Δ^9-THC	epi-bornyl-Δ^9-tetrahydrocannabinolate-C5	C32H46O4	493.33233			

Δ^9-THC	bornyl-Δ^9-tetrahydrocannabinolate-C5	C32H46O4	493.33233
Δ^9-THC	α-terpenyl-Δ^9-tetrahydrocannabinolate-C5	C32H46O4	493.33233
Δ^9-THC	4-terpenyl-Δ^9-tetrahydrocannabinolate-C5	C32H46O4	493.33233

*Additional potential phytocannabinoids that were added to the list according to their biosynthesis and decomposition pathways described in the literature.

Table S2. Correlation coefficients (R^2), limit of quantification (LOQ) and precision for the type I phytocannabinoids expressed as relative standard deviation (RSD)

Analyte	R^2	LOQ [ng/ml]	Concentration[a] [%]	Precision [%]	
				Repeatability	Reproducibility
Δ^9-THCA	0.9976	5	18.81[b]	13.08[b]	10.12[b]
CBDA	0.9996	1	12.01	6.15	6.91
Δ^9-THC	0.9979	2	0.98[b]	8.17[b]	11.04[b]
CBDVA	0.9997	0.25	0.48	6.89	7.90
CBD	0.9995	1	0.41	4.62	8.66
CBGA	0.9933	2.5	0.19	5.96	6.74
CBG	0.9997	2	0.04	4.84	5.57
CBDV	0.9996	1.25	0.03	4.54	6.41
CBC	0.9991	0.25	0.03	4.09	8.82
Δ^9-THCV	0.9999	0.25	0.02[b]	9.16[b]	9.81[b]
CBN	0.9997	1.25	0.01[b]	8.05[b]	12.25[b]
Δ^8-THC	0.9977	1.25	<LOQ	-	-
CBL	0.9997	1.25	<LOQ	-	-

[a] Average concentrations from ethanolic extractions of the same sample (n=5).
[b] Precision values quantified according to a second sample.

Table S3. Ranges of absolute phytocannabinoid contents for the 36 *Cannabis* samples

Phyto. Abbreviation	Phyto. type	Calibration curve used	**Minimum and maximum concentrations and deviations of the 36 *Cannabis* samples (Fig. 4)**					
			Min conc. [% w/w]	Min deviation of Min conc. [% w/w]	Max deviation of Min conc. [% w/w]	Max conc. [% w/w]	Min deviation of Max conc. [% w/w]	Max deviation of Max conc. [% w/w]
Δ^9-THCA*	I	-	0.2988	-	-	19.9701	-	-
Δ^9-THC*	I	-	0.0152	-	-	4.2287	-	-
Δ^9-THCA-C4	II	Δ^9-THCA	0.0009	0.0004	0.0094	0.1185	0.0515	1.2492
Δ^9-THC-C4	II	avg(Δ^9-THC,Δ^9-THCV)	0.0000	0.0000	0.0000	0.0107	0.0005	0.0121
Δ^9-THCVA	II	Δ^9-THCA	0.0021	0.0009	0.0217	0.4357	0.1892	4.5926
Δ^9-THCV*	I	-	0.0000	-	-	0.0220	-	-
Δ^9-THCOA	II	Δ^9-THCA	0.0000	0.0000	0.0000	0.0632	0.0274	0.6662
Δ^9-THCO	II	avg(Δ^9-THC,Δ^9-THCV)	0.0000	0.0000	0.0000	0.0000	0.0000	0.0000
Δ^9-THCMA	II	Δ^9-THCA	0.0000	0.0000	0.0000	0.0037	0.0016	0.0393
Δ^9-THCM	II	avg(Δ^9-THC,Δ^9-THCV)	0.0000	0.0000	0.0000	0.0000	0.0000	0.0000
CBDA*	I	-	0.0308	-	-	13.4860	-	-
CBD*	I	-	0.0000	-	-	2.0748	-	-
CBDA-C4	II	avg(CBDA,CBDVA)	0.0001	0.0001	0.0021	0.0779	0.0707	1.7171
CBD-C4	II	avg(CBD,CBDV)	0.0000	0.0000	0.0000	0.0055	0.0010	0.0247
CBDVA*	I	-	0.0001	0.0001	0.0032	1.0959	1.0959	26.6070
CBDV*	I	-	0.0000	-	-	0.0742	-	-
CBDOA	II	avg(CBDA,CBDVA)	0.0000	0.0000	0.0000	0.0166	0.0151	0.3655
CBDO	II	avg(CBD,CBDV)	0.0000	0.0000	0.0000	0.0000	0.0000	0.0000
CBDMA	II	avg(CBDA,CBDVA)	0.0000	0.0000	0.0000	0.0091	0.0083	0.2015
CBDM	II	avg(CBD,CBDV)	0.0000	0.0000	0.0000	0.0013	0.0003	0.0061
CBGA*	I	-	0.0510	-	-	1.9291	-	-

CBG*	I	-	0.0213	-	-	0.1730	-	-
CBGA-C4	II	CBGA	0.0000	0.0000	0.0000	0.0031	0.0025	0.0613
CBG-C4	II	CBG	0.0000	0.0000	0.0000	0.0002	0.0001	0.0013
CBGVA	II	CBGA	0.0000	0.0000	0.0000	0.0239	0.0198	0.4809
CBGV	II	CBG	0.0000	0.0000	0.0000	0.0030	0.0007	0.0176
CBGOA	II	CBGA	0.0000	0.0000	0.0000	0.0036	0.0030	0.0721
CBGO	II	CBG	0.0000	0.0000	0.0000	0.0001	0.0000	0.0008
CBGMA	II	CBGA	0.0000	0.0000	0.0000	0.0002	0.0002	0.0044
CBGM	II	CBG	0.0000	0.0000	0.0000	0.0002	0.0001	0.0013
SesquiCBGA	II	CBGA	0.0001	0.0001	0.0026	0.0026	0.0022	0.0526
SesquiCBG	II	CBG	0.0022	0.0005	0.0129	0.0273	0.0067	0.1622
CBCA	II	CBDA	0.0328	0.0268	0.6501	0.3132	0.2553	6.1988
CBC*	I	-	0.0050	-	-	0.1096	-	-
CBCA-C4	II	CBDA	0.0000	0.0000	0.0000	0.0033	0.0027	0.0648
CBC-C4	II	CBC	0.0000	0.0000	0.0000	0.0007	0.0001	0.0035
CBCVA	II	CBDA	0.0004	0.0003	0.0077	0.0651	0.0531	1.2889
CBCV	II	CBC	0.0000	0.0000	0.0000	0.0136	0.0027	0.0647
CBCOA	II	CBDA	0.0000	0.0000	0.0000	0.0047	0.0038	0.0929
CBCO	II	CBC	0.0000	0.0000	0.0000	0.0000	0.0000	0.0000
CBNA	II	avg(Δ^9-THCA,CBDA,CBGA) /avg(Δ^9-THC,CBD,CBG)*CBN	0.0002	0.0002	0.0164	0.0809	0.0809	5.6388
CBN*	II	-	0.0000	-	-	0.1502	-	-
CBNA-C4	II	avg(Δ^9-THCA,CBDA,CBGA) /avg(Δ^9-THC,CBD,CBG)*CBN	0.0000	0.0000	0.0000	0.0004	0.0004	0.0281
CBN-C4	II	CBN	0.0000	0.0000	0.0000	0.0006	0.0004	0.0099
CBNVA	II	avg(Δ^9-THCA,CBDA,CBGA) /avg(Δ^9-THC,CBD,CBG)*CBN	0.0000	0.0000	0.0000	0.0011	0.0011	0.0736
CBNV	II	CBN	0.0000	0.0000	0.0000	0.0011	0.0008	0.0194
CBNOA	II	avg(Δ^9-THCA,CBDA,CBGA) /avg(Δ^9-THC,CBD,CBG)*CBN	0.0000	0.0000	0.0000	0.0001	0.0001	0.0101

CBNO	II	CBN	0.0000	0.0000	0.0000	0.0001	0.0000	0.0011
OH-CBNA	II	avg(Δ^9-THCA,CBDA,CBGA) /avg(Δ^9-THC,CBD,CBG)*CBN	0.0000	0.0000	0.0000	0.0000	0.0000	0.0000
OH-CBN	II	CBN	0.0000	0.0000	0.0000	0.0009	0.0006	0.0150
CBNM	II	CBN	0.0000	0.0000	0.0000	0.0000	0.0000	0.0000
CBEA	III	avg(acids)	0.0000	0.0000	0.0000	0.0275	0.0212	0.5141
CBE	III	avg(neutrals)	0.0000	0.0000	0.0000	0.0037	0.0009	0.0211
CBEVA	III	avg(acids)	0.0000	0.0000	0.0000	0.0009	0.0007	0.0167
CBEV	III	avg(neutrals)	0.0000	0.0000	0.0000	0.0000	0.0000	0.0000
CBNDA	III	avg(acids)	0.0000	0.0000	0.0000	0.0054	0.0042	0.1016
CBND	III	avg(neutrals)	0.0000	0.0000	0.0000	0.0000	0.0000	0.0000
CBNDVA	III	avg(acids)	0.0000	0.0000	0.0000	0.0002	0.0002	0.0038
Δ8-THC*	I	Δ8-THC	0.0000	-	-	0.0004	-	-
CBL*	I	CBL	0.0000	-	-	0.0007	-	-
CBTA-1	III	avg(acids)	0.0000	0.0000	0.0000	0.0140	0.0107	0.2605
CBT-1	III	avg(neutrals)	0.0001	0.0000	0.0008	0.0711	0.0165	0.4002
CBTV-1	III	avg(neutrals)	0.0000	0.0000	0.0000	0.0005	0.0001	0.0028
CBTA-3	III	avg(acids)	0.0002	0.0002	0.0043	0.0686	0.0528	1.2814
CBT-3	III	avg(neutrals)	0.0000	0.0000	0.0000	0.0522	0.0121	0.2938
CBTV-3	III	avg(neutrals)	0.0000	0.0000	0.0000	0.0000	0.0000	0.0000
CBT-2	III	avg(neutrals)	0.0000	0.0000	0.0000	0.0104	0.0024	0.0586
329-11a	IV	avg(neutrals)	0.0000	0.0000	0.0000	0.0182	0.0042	0.1027
329-11b	IV	avg(neutrals)	0.0000	0.0000	0.0000	0.0089	0.0021	0.0499
329-11c	IV	avg(neutrals)	0.0000	0.0000	0.0000	0.0151	0.0035	0.0849
329-11d	IV	avg(neutrals)	0.0000	0.0000	0.0000	0.0026	0.0006	0.0144
373-12a	IV	avg(acids)	0.0000	0.0000	0.0000	0.0355	0.0273	0.6633
373-12b	IV	avg(acids)	0.0007	0.0005	0.0129	0.0047	0.0036	0.0879
373-12c	IV	avg(acids)	0.0000	0.0000	0.0000	0.0022	0.0017	0.0415

373-12d	IV	avg(acids)	0.0000	0.0000	0.0000	0.0064	0.0049	0.1192
327-13a	IV	avg(neutrals)	0.0000	0.0000	0.0000	0.0006	0.0001	0.0036
327-13b	IV	avg(neutrals)	0.0000	0.0000	0.0000	0.0004	0.0001	0.0022
327-13c	IV	avg(neutrals)	0.0000	0.0000	0.0000	0.0522	0.0121	0.2936
371-14a	IV	avg(acids)	0.0000	0.0000	0.0000	0.0011	0.0009	0.0208
371-14b	IV	avg(acids)	0.0000	0.0000	0.0000	0.0086	0.0066	0.1606
417-15a	IV	avg(acids)	0.0000	0.0000	0.0000	0.0073	0.0056	0.1371
373-15b	IV	avg(neutrals)	0.0000	0.0000	0.0000	0.0045	0.0010	0.0251
373-15c	IV	avg(acids)	0.0000	0.0000	0.0000	0.1718	0.1321	3.2081
357-16a	IV	avg(acids)	0.0023	0.0018	0.0438	0.0228	0.0175	0.4252
313-16b	IV	avg(neutrals)	0.0004	0.0001	0.0022	0.0630	0.0146	0.3546
361-17a	IV	avg(neutrals)	0.0000	0.0000	0.0000	0.0205	0.0047	0.1152
361-17b	IV	avg(neutrals)	0.0000	0.0000	0.0000	0.0006	0.0001	0.0035
331-18a	IV	avg(neutrals)	0.0000	0.0000	0.0000	0.1020	0.0237	0.5745
331-18b	IV	avg(neutrals)	0.0275	0.0064	0.1549	0.2615	0.0606	1.4722
331-18c	IV	avg(neutrals)	0.0000	0.0000	0.0000	0.0023	0.0005	0.0129
331-18d	IV	avg(neutrals)	0.0220	0.0051	0.1239	0.1067	0.0248	0.6009
375-19a	IV	avg(acids)	0.0015	0.0012	0.0285	0.1482	0.1140	2.7677
375-19b	IV	avg(acids)	0.0000	0.0000	0.0000	0.0018	0.0014	0.0331
375-19c	IV	avg(acids)	0.0000	0.0000	0.0000	0.0030	0.0023	0.0559

*Analytical standard
